# Longitudinal profiling of circulating tumour DNA for tracking tumour dynamics in pancreatic cancer

**DOI:** 10.1186/s12885-022-09387-6

**Published:** 2022-04-07

**Authors:** Lavanya Sivapalan, Graeme J. Thorn, Emanuela Gadaleta, Hemant M. Kocher, Helen Ross-Adams, Claude Chelala

**Affiliations:** 1 Centre for Cancer Biomarkers and Biotherapeutics, Barts Cancer Institute, Queen Mary University of London, London, EC1M 6BQ UK; 2 Centre for Tumour Biology, Barts Cancer Institute, Queen Mary University of London, London, EC1M 6BQ UK

**Keywords:** Circulating tumour DNA, Liquid biopsy, Biomarkers, Monitoring

## Abstract

**Background:**

The utility of circulating tumour DNA (ctDNA) for longitudinal tumour monitoring in pancreatic ductal adenocarcinoma (PDAC) has not been explored beyond mutations in the *KRAS* proto-oncogene. Here, we aimed to characterise and track patient-specific somatic ctDNA variants, to assess longitudinal changes in disease burden and explore the landscape of actionable alterations.

**Methods:**

We followed 3 patients with resectable disease and 4 patients with unresectable disease, including 4 patients with ≥ 3 serial follow-up samples, of whom 2 were rare long survivors (> 5 years). We performed whole exome sequencing of tumour gDNA and plasma ctDNA (*n* = 20) collected over a ~ 2-year period from diagnosis through treatment to death or final follow-up. Plasma from 3 chronic pancreatitis cases was used as a comparison for analysis of ctDNA mutations.

**Results:**

We detected > 55% concordance between somatic mutations in tumour tissues and matched serial plasma. Mutations in ctDNA were detected within known PDAC driver genes (*KRAS, TP53, SMAD4*, *CDKN2A*), in addition to patient-specific variants within alternative cancer drivers *(NRAS, HRAS, MTOR, ERBB2, EGFR, PBRM1*), with a trend towards higher overall mutation loads in advanced disease. ctDNA alterations with potential for therapeutic actionability were identified in all 7 patients, including DNA damage response (DDR) variants co-occurring with hypermutation signatures predictive of response to platinum chemotherapy. Longitudinal tracking in 4 patients with follow-up > 2 years demonstrated that ctDNA mutant allele fractions and clonal trends were consistent with CA19-9 measurements and/or clinically reported disease burden. The estimated prevalence of ‘stem clones’ was highest in an unresectable patient where changes in ctDNA dynamics preceded CA19-9 levels. Longitudinal evolutionary trajectories revealed ongoing subclonal evolution following chemotherapy.

**Conclusion:**

These results provide proof-of-concept for the use of exome sequencing of serial plasma to characterise patient-specific ctDNA profiles, and demonstrate the sensitivity of ctDNA in monitoring disease burden in PDAC even in unresectable cases without matched tumour genotyping. They reveal the value of tracking clonal evolution in serial ctDNA to monitor treatment response, establishing the potential of applied precision medicine to guide stratified care by identifying and evaluating *actionable* opportunities for intervention aimed at optimising patient outcomes for an otherwise intractable disease.

**Supplementary Information:**

The online version contains supplementary material available at 10.1186/s12885-022-09387-6.

## Background

Pancreatic ductal adenocarcinoma (PDAC) is a leading cause of cancer deaths worldwide, with few effective treatment options and a dismal 5-year survival rate of ~ 7% [1]. Systemic chemotherapy is standard care for > 80% of patients who are diagnosed with unresectable PDAC, despite the lack of clinically meaningful survival benefits [1]. The recent use of potent combination chemotherapies has delivered modest improvements in survival outcomes for a proportion of unresectable patients, although clinical applications are currently limited by toxicity [2]. Even in patients who undergo surgery, early recurrences (within 6 months) occur in 28% of cases, attributed to the presence of micro-metastatic disease at the time of resection [3]. To improve treatment efficacy and survival outcomes in PDAC, better stratification of patients and monitoring of tumour burden and responses to treatment is essential.

Tumour-derived genetic alterations have been identified and analysed through fragments of circulating tumour DNA (ctDNA) in peripheral blood, allowing for a minimally invasive approach to tumour sampling for monitoring strategies [4, 5]. ctDNA can provide aggregate information on multiple clonal subsets within primary tumours and metastases, presenting significant advantages over invasive single-region tissue biopsies [6–8]. However, the low fractional abundance of ctDNA in patients with PDAC has presented a significant challenge for the analysis of mutation profiles [9–11]. Most previous studies have focussed on patients with advanced disease and a higher anticipated ctDNA burden, using droplet digital PCR (ddPCR) to detect *KRAS* variants or targeted sequencing of a small number of key hotspot mutations [5, 12, 13]. These strategies have failed to adequately capture the extent of inter-tumoural genetic heterogeneity between PDAC tumours, resulting in significant variability between reported ctDNA detection rates (< 12% up to 100%) [14]. This is likely to be associated with the effects of sampling variation, which can impair detection sensitivities in heterogenous disease and when the number of copies of mutant DNA in patient plasma is low [15].

In contrast, broader genomic interrogation of patient-specific ctDNA variants using exome sequencing may provide a more accurate representation of circulating tumour burden in individual PDAC patients [15–19]. Here, we investigate the utility of longitudinal exome sequencing in an exploratory cohort of 7 patients with localised, locally-advanced and metastatic PDAC. Using an optimised analytical pipeline, we identify and track patient-specific ctDNA mutations from baseline (pre-treatment) throughout follow-up, in samples taken at clinically determined intervals after patients received treatment with surgery and/or chemotherapy (2-year window—until death (*n* = 4) or last follow-up (*n* = 3)). Our results demonstrate that exome sequencing of plasma can enable personalised monitoring of ctDNA burden and clinically actionable mutation profiles in response to treatment and/or disease progression.

## Methods

### Patients and sample collection

Blood and tumours from patients with PDAC were obtained with written informed consent and processed by the Barts Pancreatic Tissue Bank (www.bartspancreastissuebank.org.uk, Research Ethics Committee reference 13/SC/0592, project references 2015/05/QM/CC/ctDNA, 2017/06/QM/CC/C/Blood&Tissue and 2018/15/QM/CC/E/Blood). We evaluated baseline plasma from 3 chronic pancreatitis (CP) cases, as benign controls for analysis of ctDNA variants. Plasma from *n* = 5 healthy controls was obtained for comparative analysis of total cell-free DNA (cfDNA); these were not sequenced due to very low total yields.

### Sample processing and DNA extraction

Multiple vials of whole blood were drawn at each clinic for all patients for a suite of indicated tests, including our ctDNA analysis and CA19-9 levels (tested by hospital Biochemistry). For the former, whole blood samples were collected in either 10 mL Vacutainer K3EDTA tubes (BD) or in RUO Cell-Free DNA Collection Tubes (Roche) and processed for plasma and buffy-coat isolation within 2 h of collection through 2 centrifugation steps, each performed at room temperature for 10 min at 1,600 g. cfDNA was extracted from 1.5 mL-3 mL plasma using the QIAamp MinElute ccfDNA kit (Qiagen, manufacturer’s instructions), for immediate analysis. DNA from fresh-frozen bulk tumour sections and buffy coat was extracted using the DNeasy Blood and Tissue kit (Qiagen, manufacturer’s instructions) and stored at -80^0^C.

### Sequencing of tumour and plasma DNA

Plasma libraries were prepared from up to 10 ng cfDNA using Rubicon ThruPLEX Plasma-Seq kits. Exome capture of plasma libraries was performed using SureSelect XT2 v6.0 human all exon (Agilent) kits with the addition of i5 and i7 xGen Universal Blocking Oligos (Integrated DNA Technologies), in line with the manufacturer’s recommendations for compatibility with ThruPLEX libraries. Enriched libraries were quantified (Qubit) and pooled for sequencing on NovaSeq 6000 (Illumina) to 1000X target depth. Plasma libraries from patients 45 and 95 (P1-P4 from patient 45 and P1-P4 from patient 95) were pooled and sequenced on HiSeq 4000 (Illumina) to 500X target depth. Tumour and germline (buffy coat) DNA samples were sonicated to a target fragment size of ~ 200 bp. Sequencing libraries were prepared from up to 100 ng of sheared germline DNA using HSQ SureSelect XT2 Reagent kits (Agilent), according to the manufacturer’s recommendations. Germline DNA libraries were pooled for exome enrichment using SureSelect XT2 v6.0 human all exon kits (Agilent), as described above, and sequenced on NovaSeq 6000 (Illumina) to 100X target depth. Sequencing of both plasma and germline DNA libraries was performed at the CRUK Cambridge Institute (Genomics Core).

Whole genome sequencing was performed on tumour samples. Library preparation of up to 1 µg sheared tumour DNA (using TruSeq nano DNA sample preparation kits (Illumina)), sequencing, alignment and variant calling was performed by Edinburgh Genomics. Tumour sequencing from patient 28 failed quality control and was therefore not evaluated in this study.

### Bioinformatic analysis of sequencing data

Paired-end reads were aligned to the hg38 human reference genome using BWA-MEM (v0.7.15). Duplicate reads were marked using Picard (from Genome Analysis Tool Kit v4.1.3.0) and removed prior to variant calling for tumour and germline samples. Duplicate reads were left unmarked for plasma analysis. Base quality score recalibration and indel realignment was performed using GATK v4.1.3.0.

Variants were then called per patient, using samtools (v1.9) mpileup, and VarScan (v2.4.3) in multi-sample mode, with a minimum coverage of 3 reads with one read on each strand for a variant to be called in plasma, and annotated using ANNOVAR. Mutations supported by at least 1 read were called in plasma if they were also present in a matched tumour sample with coverage of ≥ 3 reads. Called variants were filtered to remove any mutations that were absent in the COSMIC91 database but with a corresponding identifier in the dbSNP database. Variants were also filtered on exonic function, to remove mutations with ‘synonymous’ or ‘unknown’ classifications. Only variants with an alternate allele base quality score ≥ 25, and no alternate reads in either matched germline DNA (at a site covered ≥ 20x) or plasma DNA from CP cases, were retained.

### Filtering of plasma variants

To enrich for candidate ctDNA mutations and minimise the number of false-positive calls, multi-allelic variants were removed and only mutations with a single alternative genotype across serial plasma from each patient retained. Alternate allele frequencies for plasma variants were assessed across normal populations from the 1000 Genomes Project (1000G), the Genome Aggregation Database (gnomAD) and Haplotype Map (HapMap) project using SNPnexus (http://www.snp-nexus.org/) [20]. Plasma variants with reported mutant allele frequencies (> 0%) across these populations were flagged. Known or predicted (TIER 1 and TIER 2) driver mutations in plasma, and actionable mutations of relevance for targeted treatment, were annotated using the Cancer Genome Interpreter (CGI) function in SNPnexus. To adjust for problematic genomic regions and increase the specificity for detection of true mutations, the hg38 ENCODE blacklist (https://github.com/Boyle-Lab/Blacklist) [21] was applied to filtered patient-specific variants. The presence of false positives arising from systematic artefacts (e.g. strand bias) was also excluded using the FPfilter accessory script (https://github.com/genome/fpfilter-tool), which was run on all candidate ctDNA mutations [22]. A summary of the complete analytical pipeline is shown in Supplementary Fig. 2.

### Estimation of copy number alterations in tumour and plasma

Genome-wide copy number alterations were determined using ichorCNA (v0.3.2), with BAM files from paired tumour-germline or plasma-germline samples as input. WIG files with non-overlapping 1 Mb bins across chromosomes were generated from matched WGS (tumour)/WES (plasma) and normal (PBMC-derived) BAM files for each patient, using the ‘readCounter’ function from HMMCopy. Only variant reads with a mapping quality ≥ 20 were used to generate WIG files. Aligned reads were counted based on overlap within each bin and centromeres filtered using chromosomal gap coordinates. Read counts for each bin were normalised for GC content and mappability biases, using a LOESS regression curve fitting applied to autosomes. Pathology-derived tumour cellularity estimates were used to inform copy number predictions for tumour samples. Tumour fractions were estimated in plasma using the intrinsic purity prediction function of ichorCNA. The global optimum for estimated tumour fraction in plasma was initialised according to expected normal cell contamination values (in the range of 0.2, 0.35, 0.5, 0.65, 0.8, 0.9, 0.99), and analyses run on ‘clonal-only’ mode. Copy number estimates from ichorCNA were verified using CopyWriteR (v2.0.6) and Sequenza (v3.0.0).

### Identification of enriched mutational signatures in tumour and plasma

Mutational signatures were analysed using the R package deconstructSigs (v1.8.0), alongside the Bioconductor library BS.genome.Hsapiens.UCSC.hg38.

### Analysis of pathway enrichments

Enriched gene signalling pathways were analysed using ClueGO and the R package ReactomePA (v1.16.2). A hypergeometric model was used to determine whether the number of selected genes associated with each pathway in the Reactome database was greater than expected by chance.

### Identification of kataegis events in tumour and plasma

Rainfall plots were generated using the R package KaryoploteR (v1.16.0) [23]. A positive kataegis event was defined as the presence of 6 or more mutations with an average inter-mutational distance of ≤ 1000 bp. Quantitative analysis of kataegis events was performed using R packages ClusteredMutations (v1.0.1), MAFtools (v0.9.3) and Seqkat (v0.0.8) [24]. The minimum hypermutation score used to classify windows in the sliding binomial test as significant during Seqkat analysis was 5, the maximum log_10_(inter-mutational distance) for SNVs to be grouped into the same kataegis event was 4 and the minimum number of mutations required within a cluster to be classified as kataegis was 6.

### Inferring clonal structures and evolutionary trajectories in ctDNA

Filtered lists of ctDNA variants were derived for 4 patients with ≥ 3 serial plasma samples, using the pipeline described above. Reference and alternate reads for each variant per patient per plasma sample were clustered using Absence Aware Clustering (https://github.com/raphael-group/Absence-Aware-Clustering), based on similar variant allele fractions. Clustered mutations were run through CALDER [25], which returned clonal determinations and prevalence per clone at each plasma timepoint. Results were visualised using the timescape package in R (v1.14.0), with the clonal trees outputted by timescape redrawn.

## Results

### Tumour-specific somatic mutations are detected in plasma using exome sequencing

**Fig. 1:**
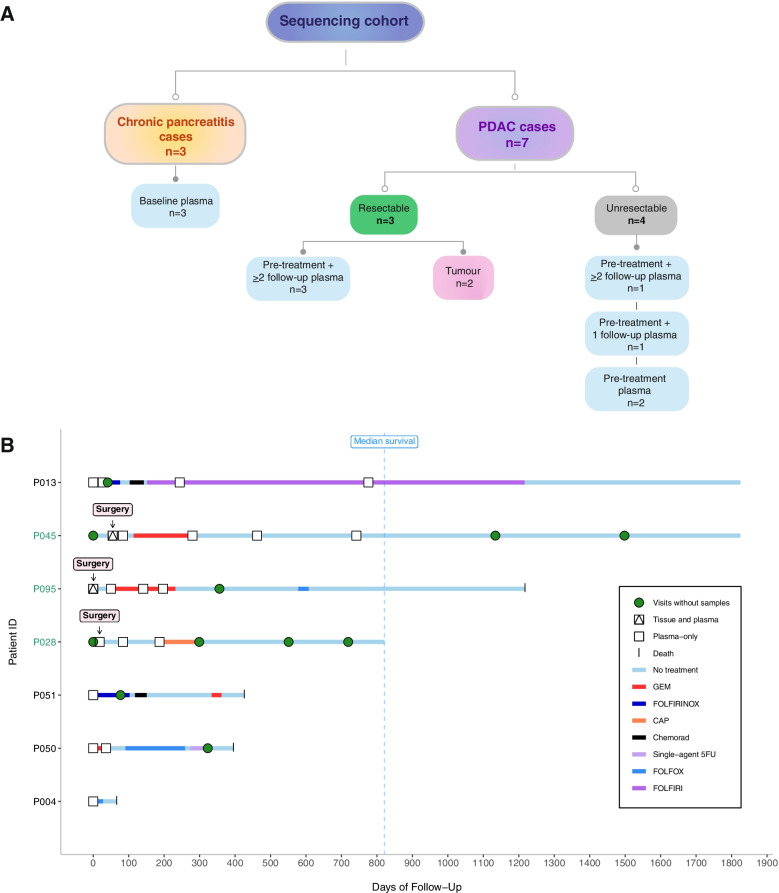
Summary of patients and samples for sequencing.** A** Outline of samples available for exome sequencing from PDAC and chronic pancreatitis (CP) control cases. **B** Clinical timelines including survival and treatment periods for sequenced PDAC patients. 5FU, 5-Fluorouracil; CAP, Capecitabine; Chemorad, Chemoradiation; GEM, Gemcitabine

We retrospectively profiled, in a blinded manner, 20 blood samples from 7 patients with histologically confirmed PDAC; including 3 patients who underwent surgical resection (cases 45, 95, 28) and 4 patients with advanced unresectable disease (cases 04, 13, 50, 51). Blood samples from 3 chronic pancreatitis (CP) and 5 healthy control (HC) cases were included as benign comparators (Fig. 1, Supplementary Fig. 1). Serial blood samples were available from 5 patients, of whom 4 cases had ≥ 3 serial samples which were collected at clinically determined intervals, separated by consecutive lines of therapy (Fig. 1). Clinical characteristics of the study patients are summarised in Supplementary Table 1. Overall concentrations of cfDNA were higher amongst patients with PDAC compared to CP and HC cases (who had undetectable cfDNA levels) (Supplementary Fig. 1a). A trend towards higher cfDNA levels was also observed amongst unresectable PDAC patients compared to resectable cases, although this was not statistically significant (Supplementary Fig. 1a, b).

**Fig. 2:**
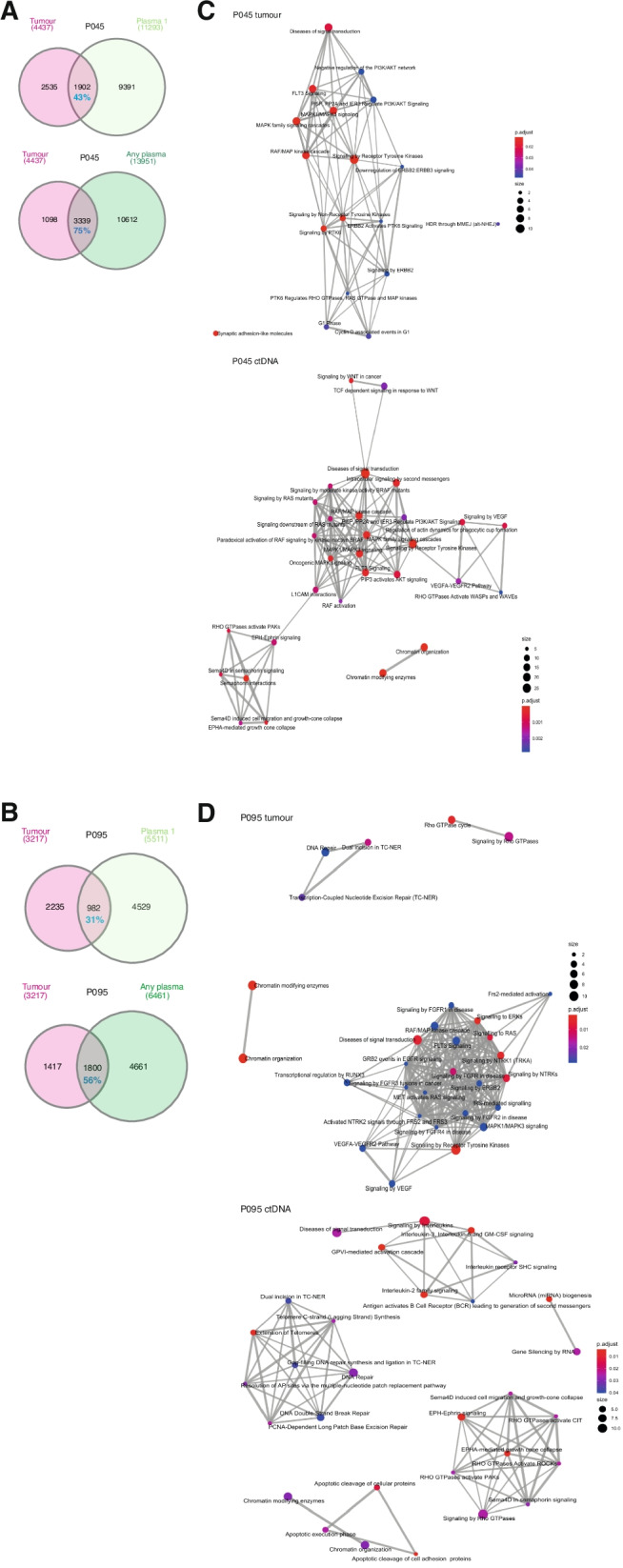
Comparison between somatic mutations in tumour and matched plasma from patients 45 and 95. Overlaps between somatic mutation calls in tumour and baseline pre-treatment (P1) plasma *(top)*, and combined plasma (P1-P5/P4) from baseline plus follow-up sampling *(bottom)* in each patient, are shown in **A** and **B**. Comparisons were used to inform the development of our custom analysis pipeline, for the identification of candidate ctDNA mutations in plasma. Enriched gene signalling pathways (Reactome) observed in tumour tissues and ctDNA variants from combined plasma samples are shown in **C** and **D**

Somatic mutations in tumour and time-matched pre-treatment (P1) plasma from 2 resectable patients were profiled using our custom variant analysis pipeline (summarised in Supplementary Fig. 2; *see* *Methods*), demonstrating a variant overlap of 43% and 31% of calls within tumour respectively, which increased to 75% and 56% upon the comparison of tumour with *combined* all time-point plasma variants (Fig. 2a, b, Supplementary Fig. 1c-h**)**. Most overlapping mutations occurred at variant allele fractions < 10% in both tumour and plasma (Supplementary Fig. 1c, d). No significant associations were identified between the variant allele fractions or coverage of mutations in plasma and overlap with tumour tissues (Supplementary Fig. 1c-h).

To evaluate the utility for size selection to improve the sensitivity for ctDNA detection in patients, fragmentation profiles were inferred from plasma sequencing reads containing mutant and wild-type alleles at target loci for ctDNA. A 167 bp modal fragment size was observed across mutant and wild-type fragments from most patients, indicating limited value for selective analysis based on modal sizes (Supplementary Fig. 1i).

Pathway analyses revealed enrichment of multiple tumour-associated pathways [26–28] across ctDNA from patients, including RAS/MAPK signalling, chromatin modification, axonal guidance and DNA damage repair (DDR) (Figs. 2c, d, Supplementary Fig. 3**)**. Combined analysis of somatic ctDNA variants identified across the study cohort revealed higher mutation loads in ctDNA compared to sequenced tumours, with the highest ctDNA mutation loads observed in advanced unresectable cases **(**Supplementary Fig. 4, Supplementary Table 2, Fig. 4b).

### Tumour structural variations and localised hypermutation events are captured in plasma through ctDNA

**Fig. 3:**
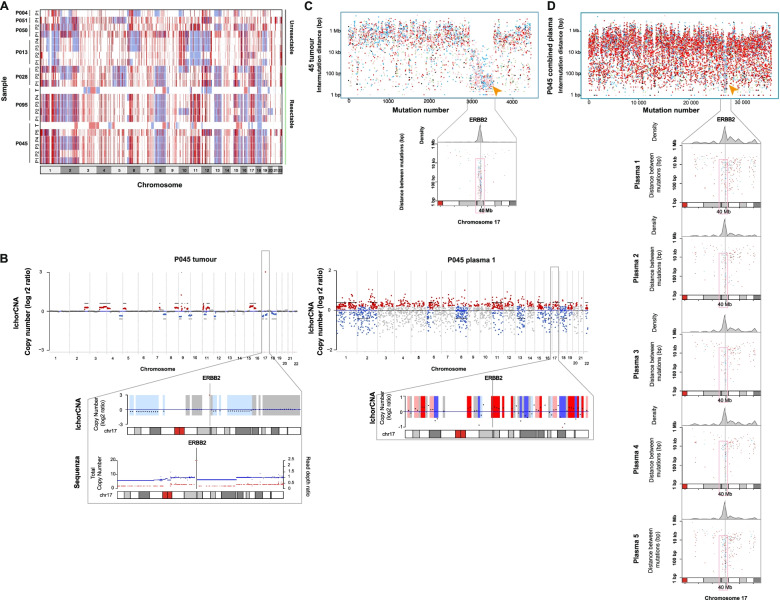
Analysis of somatic copy number alterations and localised hypermutation in tumour and plasma. Absolute copy number calls from tumour and plasma samples are shown in **A**. Gains in overall copy number are highlighted in *red* and losses of copy number are shown in *blue.* Genome-wide somatic copy number calls in tumour *(left)* and matched baseline (pre-treatment) plasma *(right)* from patient 45 are displayed in **B.** Amplifications and copy number gains at the *ERBB2* locus on chromosome 17 were observed in both tumour and plasma from this patient. **C, D** Rainfall plots showing the distribution of single somatic substitutions in tumour (**C**) and combined plasma (**D**) from patient 45, with *arrows* highlighting the presence of a unique kataegis region on chromosome 17 co-localising with *ERBB2* amplification. This region was enriched for T > G substitutions and contained *ERBB2* driver mutations in tumour, which were also detected in ctDNA. Inter-mutation distance is presented on the vertical axis and the number of mutations in each sample on the horizontal axis

Shared regions of copy number (CN) gain and loss were observed in matched tumour-plasma samples from patients 45 and 95 across chromosomes 11, 15, 17 and 18 (Fig. 3a, b). This included focal amplification of *ERBB2* (chromosome 17) in tumour from patient 45, identified as amplifications (P1-P4) and gains in copy number (P5) across matched serial plasma (Fig. 3a, b). Multiple plasma-specific SCNAs were also identified in each patient, resulting in a greater overall number of CN calls in plasma compared to tumour tissues (*P* < 0.0001) (Fig. 3a, Supplementary Fig. 5a**).** Furthermore, combined analysis of all study patients indicated a significant loss of copy number in both tumour (93% of all chromosome 18 tumour CN calls) and 12/20 plasma samples on chromosome 18 (82% of all chromosome 18 plasma CN calls) (Fig. 3a, Supplementary Fig. 5b**)**. In contrast, only CN gains were identified in plasma on chromosomes 3, 4, 7, 9 and 14 (Fig. 3a, Supplementary Fig. 5c**)**. Focal plasma gains were identified on chromosome 12p, at the *KRAS* locus, in one patient (patient 04) with multiple liver metastases at diagnosis alongside primary lesions in the pancreatic tail. CN gains at this region were concurrent with somatic *KRAS* (p.G12D) mutations in ctDNA and were verified using three independent CN calling tools (Supplementary Fig. 5d).

In addition to the observed CN changes, all patients displayed evidence of localised hypermutation, *kataegis*, in tumour and/or plasma (Supplementary Fig. 6a). Most recurrent kataegis events in plasma displayed an enrichment for C > T substitution variants (Supplementary Fig. 6a), except for a unique region identified on chromosome 17 in patient 45, a rare long-term (> 5 years) survivor, which showed a pronounced increase in T > G somatic substitutions across tumour and serial plasma (P1-P5) (Fig. 3c, d). This kataegis locus contained *ERBB2* driver variants, which were detected in both tumour and ctDNA, and co-localised with *ERBB2* amplification and copy number gains described previously (Fig. 3c, d). Hypermutation events co-localised with *ERBB2* amplification were not identified in TCGA and ICGC PDAC cohorts (https://dcc.icgc.org/releases/release_28/Projects/, Supplementary Fig. 6b, c), suggesting the patient-specific nature of this observed tumour event.

### ctDNA variants with potential therapeutic actionability are trackable over the course of treatment in patients

**Fig. 4:**
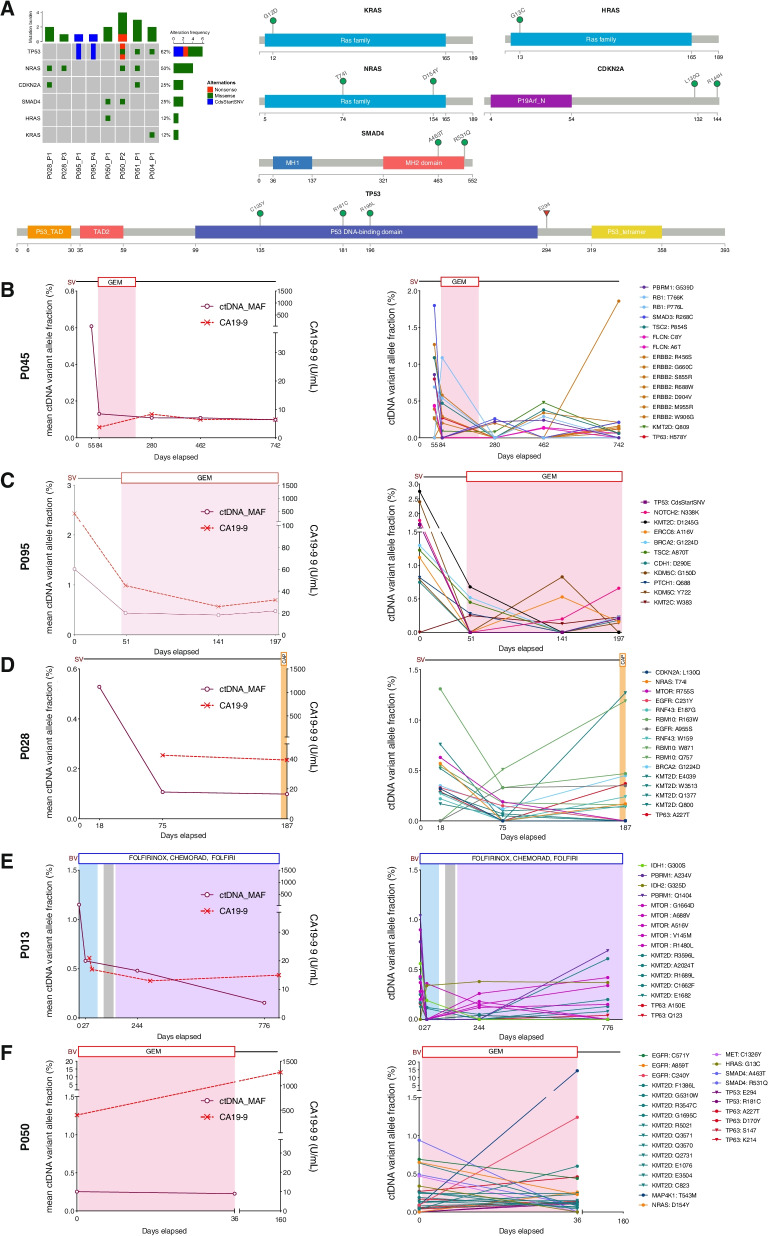
Identification of longitudinally trackable driver mutations in ctDNA.** A** Oncoprint showing patients with ctDNA mutations in PDAC drivers *(KRAS, TP53, SMAD4, CDKN2A)* and known RAS family genes *(NRAS, HRAS)* in plasma. The percentage of altered cases is displayed to the *right*. Lollipop plots displaying the mutations detected in ctDNA are shown alongside the oncoprint. **B-F** In patients with multiple plasma samples, the mean mutant allele fraction (MAF) was calculated for all mutation loci in ctDNA (patient-specific plus ctDNA variants in known PDAC drivers), at each timepoint*.* Available measurements of CA19-9 across serial timepoints for each patient are also shown*.* Examples of patient-specific ctDNA mutations observed in each case are displayed on the *right* (missense variants *(circles)*, nonsense variants *(triangles)*, CdsStartCNV variants *(squares)*)*.* In two patients, temporal heterogeneity between ctDNA mutations in *RAS* and *IDH* genes was detected **E, F**. CdsStartCNV; single nucleotide variant at coding start; CAP, Capecitabine; CHEMORAD (CAP), Chemoradiation (with Capecitabine); GEM, Gemcitabine

Longitudinal analysis of mutated genes in ctDNA highlighted multiple patient-specific variants with potential for clinical actionability. Among the variants identified in ctDNA were missense and nonsense mutations within known PDAC driver genes: *KRAS* (p.G12D), *TP53* (p.E294, p.R181C, p.R196L, p.C135Y), *SMAD4* (p.A463T, p.R531Q) and *CDKN2A* (p.L130Q, p.R144H) (Fig. 4a). Patient-specific ctDNA variants were also identified within alternative cancer drivers, including *NRAS*, *HRAS*, *TP63, MTOR, ERBB2, EGFR, PBRM1, KMT2D* and *RNF43* (Fig. 4b-f). Most variants were trackable across ≥ 2 serial plasma samples from individual patients, with trends in variant allele fractions that were correlated CA19-9 measurements and/or changes in clinically reported disease burden (Fig. 4b-f). Notably, in patient 13, dynamic changes in ctDNA levels preceded alterations in CA19-9 measurements (Fig. 4c, e). In 2 patients (patients 13 and 50), temporal heterogeneity was identified between altered driver genes in pre-and post-treatment ctDNA, with baseline variants in *HRAS* (p.G13C) and *IDH*1 (p.G300S) declining to undetectable levels following chemotherapy treatment in each case (Fig. 4e, f). These changes coincided with the emergence of new missense mutations in *NRAS* (p.D154Y) and *IDH2* (p.G325D) across post-treatment follow-up plasma from each patient (Fig. 4e, f).

**Fig. 5:**
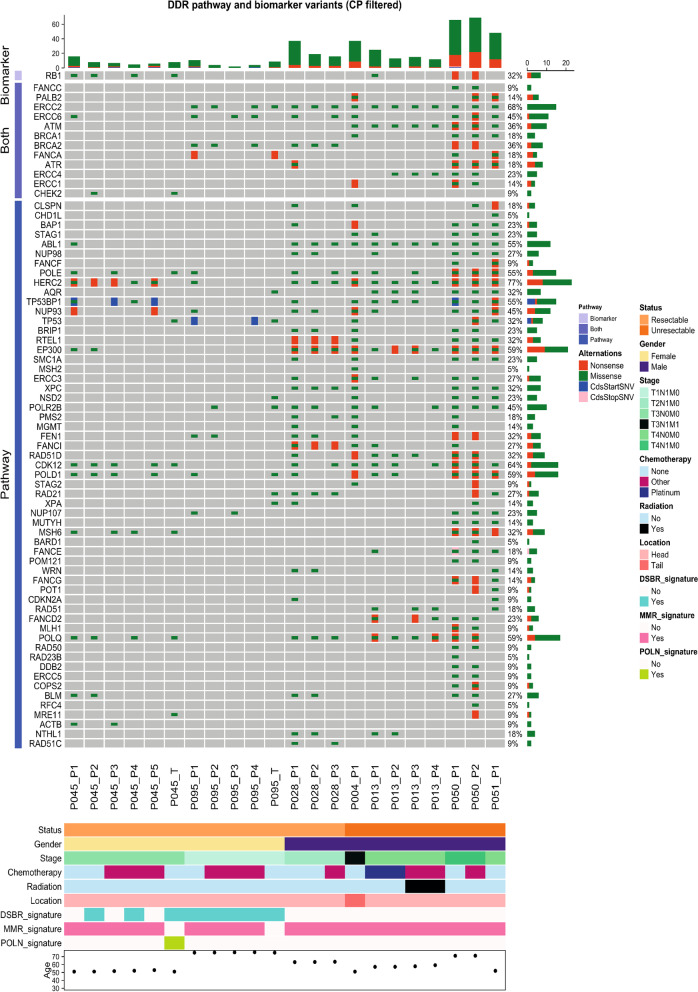
Identification of ctDNA variants with potential therapeutic actionability. Oncoprint showing mutated DNA damage repair (DDR) genes in ctDNA that were either predicted to confer response to platinum chemotherapy and/or PARP inhibition through in silico predictions (Cancer Genome Interpreter) *(Biomarkers)* or were identified within known DDR signalling pathways (Reactome) *(Pathways)*. The percentage of altered cases is displayed to the *right*. Clinical characteristics of the cohort and enrichments for COSMIC mutational signatures associated with DDR, are shown on the *bottom* panels. Post-treatment plasma samples collected following platinum or other chemotherapies and/or radiation therapy, are indicated. DSBR, double strand break repair; MMR, mismatch repair; POLN, polymerase ν (nu) hypermutation

In silico functional predictions of ctDNA variants identified across the study cohort revealed a total of 335 mutations that had either been previously reported as candidates for therapeutic targeting or were predicted to confer therapeutic utility, including 75 DNA damage-associated variants for which polyadenosine-diphosphate-ribose polymerase (PARP) inhibitor or platinum chemotherapy treatment was indicated (Fig. 5, Supplementary Fig. 7a, Supplementary Table 3**)**. We detected a further 514 ctDNA mutations within signalling pathways associated with defective DNA damage repair (DDR), which amounted to a total of 188 DDR mutations that were trackable across ≥ 2 serial plasma (Fig. 5). This included mutations in *BRCA1*, *BRCA2* and *PALB2* across five patients (04, 45, 50, 51, 95) (Fig. 5). Enrichments for 9 mutational signature classes were also observed across sequenced patients, including 3 associated with known mechanisms of genomic instability: double strand break repair (DSBR) *(COSMIC signature 3)*, defective mismatch repair (MMR) *(COSMIC signatures 6, 15, 20, 21, 26)* and hypermutation associated with polymerase ν (POLN) *(COSMIC signature 9)* [29]. (Fig. 5, Supplementary Fig. 7b**)**. Patients 45 and 95 both displayed enrichments for the BRCA-associated DSBR signature across tumour and matched plasma (Fig. 5, Supplementary Fig. 7b**)**. In patient 45, DSBR signature enrichments were concurrent with enrichments for POLN-mediated somatic hypermutation (Fig. 5, Supplementary Fig. 7b**)**. Signatures indicative of defective MMR in plasma were identified across all patients (Fig. 5, Supplementary Fig. 7b**)**.

### Ongoing clonal evolution is evident through ctDNA from serial plasma in PDAC

**Fig. 6:**
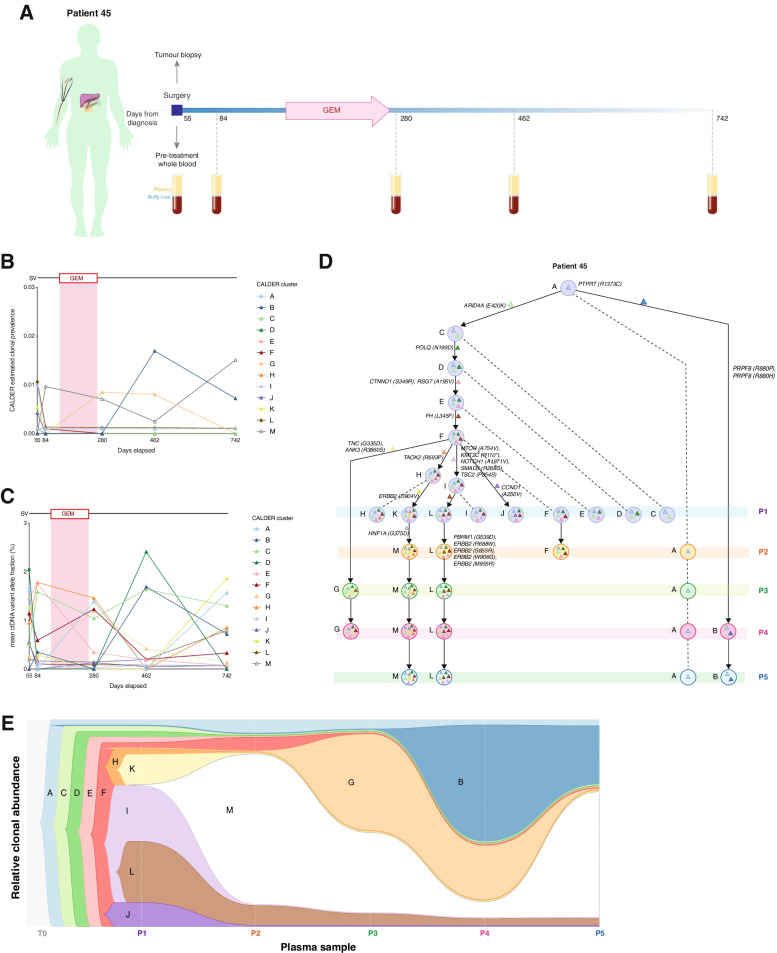
Analysis of clonal evolutionary trajectories in ctDNA from patient 45.** A** Clinical timeline for patient 45 showing treatment dates for primary tumour resection, adjuvant chemotherapy (gemcitabine) and sampling timepoints, as days from initial diagnosis. **B** Scatterplot showing the estimated prevalence of inferred clones in ctDNA, across sampled timepoints. **C** Longitudinally observed phylogenetic tree showing the predicted evolutionary trajectories of individual ctDNA clones. *Coloured triangles* represent mutations unique to each respective clone. Examples of unique driver mutations acquired in individual clones are shown on the tree. **D **Clonal diagram of the tree structure from (D) showing differences between estimated clonal proportions across sampled timepoints. GEM, Gemcitabine

Finally, we investigated whether clonal proportions and longitudinal evolutionary trajectories could be inferred from low frequency ctDNA variants in PDAC, by applying longitudinal constraints to phylogeny inferences. Patient-specific heterozygous ctDNA mutations were clustered according to similar variant allele fractions and ancestral relationships between observed clones at each sampled timepoint determined in 4 cases with ≥ 3 serial plasma. (Fig. 6, Supplementary Fig. 8). The estimated prevalence of ‘stem clones’, identified as mutation clusters with the highest predicted clonal abundance in each patient, were higher in unresectable patient 13, compared to resectable cases 28, 45 and 95.

Relative clonal abundances from ctDNA decreased after treatment in all 4 patients, consistent with CA19-9 measurements (Fig. 6, Supplementary Fig. 8**)**. Evidence of ongoing subclonal evolution was also identified from ctDNA in patients 13 and 45, with the emergence of new subclonal populations following chemotherapy treatment, coinciding with changes in genomic profiles (Fig.6, Supplementary Fig.8**)**. In patient 45, a reduction in the estimated prevalence of 8/10 ctDNA clones (clones C, D, E, H, I, J, K, L) observed at baseline pre-surgery (P1) (day 55) sampling was detected post-surgery (P2) (day 84) (Fig. 6). The prevalence of these clones remained consistently low throughout post-treatment follow-up samples (from P3 (day 280) to P5 (day 742)), in accordance with clinical reports of a significant reduction in disease burden after surgery and adjuvant Gemcitabine treatment (Fig.6). This included clone L, which was predicted to have the greatest prevalence at baseline sampling in patient 45 and contained *ERBB2* driver mutations (p.R688W, p.S855R, p.W906G, p.M955R) associated with the unique region of kataegis described previously (Figs.3, 6). We also detected the emergence of new ctDNA clones (B, G, M) throughout follow-up samples (P2-P5), which were characterised by acquired driver mutations in *PRPF8* (p.R880P, p.R880H), *TNC* (p.G335D), *ANK3* (p.R3860S) and *HNF1A* (p.G375D) (Fig.6).

Similarly, in patient 13, reductions in the estimated prevalence of 9/10 observed baseline clones (clones C, F, G, H, L, M, N, O, P) was detected between P1 (day 0) and P3 (day 244) sampling, following FOLFIRINOX, chemoradiation (Capecitabine) and initial FOLFIRI treatment, consistent with CA19-9 trends (Supplementary Fig. 8g-i). Of these, a minor increase in the relative abundance of 5 clonal populations was observed between P3 and P4 (day 776), after further continuation of FOLFIRI treatment (Supplementary Fig.8g-i). This included clones C (*ARID5B* p.A829T, *PTCH1* p.R13008*), H (*TNPO2* p.D319G), L (*MTOR* p.G1664D, p.A688V, *WNT5A* p.C240Y), N (*NCOR2* p.I796L) and O (*POLQ* p.Q2030*, *SVEP1* p.C340Y) (Supplementary Fig.8g-i). Novel ctDNA clones containing activating driver mutations also emerged throughout on-treatment follow-up samples (P2-P4) from patient 13, including clones D (*GATA3* p.F234S, *MAP3K11* p.R729P), I (*SRGAP3* p.R877W) and Q (*POLE* p.R1793M, *ARID5B* p.K1027R) (Supplementary Fig.8).

## Discussion

Recent advances in targeted ctDNA analysis have shown promise for tumour monitoring in patients with solid tumours, but these have had limited utility in cancers with high inter-tumoural heterogeneity, such as PDAC [14]. In this proof of principle study, we have demonstrated that exome sequencing of serial plasma and matched germline DNA can enable the characterisation of patient-specific ctDNA variants and tracking of actionable molecular alterations in both the early and advanced disease settings. Importantly, this included the identification of informative ctDNA alterations in patients who did not have matched tumour tissues available for sequencing. These findings indicate useful applications for a personalised approach to ctDNA analysis and longitudinal monitoring in PDAC, which has the potential to improve overall detection sensitivities compared to targeted profiling of hotspot regions or a panel of recurrent cancer genes.

We have developed and optimised a custom analytical pipeline for the identification of candidate ctDNA mutations in plasma, without the need for matched tissue genotyping, and have tested this pipeline in an exploratory cohort of 7 PDAC patients representing localised, locally-advanced and metastatic disease. Comparisons between ctDNA mutations identified using our pipeline and tumour genotyping revealed tumour-derived somatic variants, actionable alterations and pathway enrichments in plasma, where longitudinal sampling improved the characterisation of tumour mutation profiles. We also observed an increase in overall mutational burdens in ctDNA, compared to tumour tissues, indicative of the collective influence of ctDNA fragments shed from multiple different tumour clones (irrespective of anatomical biases), which are likely to capture a larger proportion of a given tumour genome, compared to a single-region biopsy specimen [30]. Whilst discrepancies were observed between a proportion of tumour and plasma variant calls in patients 45 and 95, consistent with previous reports from exome-wide ctDNA analyses [31, 32], these are likely to be attributed to the stochastic nature of ctDNA release and dynamics in peripheral blood. Observed overlaps may be further influenced by presence of localised resectable disease, which is associated with lower overall levels of ctDNA shedding [5, 6, 33, 34], in both patients with matched tumour samples available in this study. In contrast, ctDNA mutational burdens and estimated clonal prevalences were highest amongst unresectable patients from our exploratory cohort, consistent with an elevated disease burden [11].

An increase in total number of SCNAs was also detected across plasma, consistent with ctDNA shedding from multiple spatially distinct tumour clones, in addition to the larger and more significant number of normal cfDNA shedding cells, which can reflect genomic evolution with respect to SCNAs in normal tissues and white blood cells as a result of clonal haematopoiesis (CHIP) [30]. As total copy number loads in exome-captured plasma data can also be influenced by the increase in signal–noise ratios resulting from the sparsity of exonic regions and biases introduced during hybrid capture, plasma SCNAs were analysed using a combination of 3 CNV calling tools to confidently identify altered regions [31, 35]. This revealed multiple tumour-associated SCNAs in plasma, with value for the assessment of prognosis in patients. This included concurrent *KRAS* copy number gains and somatic mutations previously associated with poor prognosis in one patient (patient 04), who presented with metastatic liver lesions and displayed the poorest overall survival (< 70 days) amongst our cohort [13]. These findings were consistent with recent associations between liver metastases and *KRAS* variant allele fractions in ctDNA [5, 36]. Amplification of *ERBB2* was observed in tumour from another patient (patient 45), captured through regions of altered copy number in plasma, at a lower amplitude consistent with the low fractional abundance of ctDNA amongst non-tumour cfDNA [37]. *ERBB2* amplification occurs in ~ 2% of PDAC tumours and may outline a suitable sub-population for targeted treatment with anti-ERBB2 therapies [38]. CNVs in this region also co-localised with a unique kataegis locus, which was detected independently in both tumour and matched plasma from this patient. This region displayed a substitution profile consistent with previous reports of a rare alternative kataegis signature observed in ~ 0.9% of breast cancers [39] and characterised by T > G and T > C mutations, predominantly at NTT and NTA sequences (where N could be any base C, G, A or T) [39]. This distinct substitution pattern most closely resembles COSMIC mutational signature 9, previously observed in B lymphocyte neoplasms [40] and attributed to polymerase η (eta) activity [39]. Recently, D’Antonio et al. (2016) reported an upregulation of *ERBB2* expression in breast cancer patients who harboured similar chromosome 17 kataegis events; these patients also had an extended survival, suggesting prognostic value for kataegis profiling in solid tumours [41]. Although this distinct kataegis event was only identified in a single patient from our cohort, we highlight the unique clinical profile of patient 45, who has an overall survival exceeding 5 years from initial diagnosis. Clinical reports of stable disease have been recorded throughout recent follow-up visits for this patient, who we continue to monitor. The enrichment for focal events on chromosome 17 in this patient was consistent with previous reports of a *‘locally rearranged’* subtype of PDAC tumours, characterised by significant focal structural aberrations on one or two chromosomes [26]. The absence of chromosome 17 kataegis events co-localising with *ERBB2* amplification in TCGA and ICGC PDAC tumours was further comparable with the molecular heterogeneity of PDAC [42] and suggests this phenomenon may only present in a small sub-population of patients.

However, as a relatively new measure of genomic instability, hotspots of kataegis events in PDAC tumours are yet to be defined, presenting a challenge for de novo identification and analysis of most events in plasma that are not characterised by a distinct substitution profile. Further investigation into the genome-wide distribution and pattern of mutations within kataegis regions across larger PDAC tumour and matched plasma cohorts is essential to determine the biological and/or clinical significance of observed kataegis foci, and to evaluate potential associations with tumour characteristics and patient survival.

We then analysed the landscape of mutated genes across ctDNA, to determine whether variants within biologically and clinically relevant genes for PDAC pathogenesis could be tracked over time and following treatment in patients. Whilst the majority of ctDNA mutations were patient-specific, core signalling pathways and groups of therapeutically relevant genes, including *IDH* family genes, recently highlighted as promising targets for molecular therapy in PDAC in the Know Your Tumour Project (Pancreatic Cancer Action Network) [43], were frequently affected across the cohort. Only a small proportion of observed ctDNA mutations were within the four established PDAC drivers *(KRAS, TP53, CDKN2A, SMAD4)*, with most variants targeting alternative driver genes with relevance for tumour development and/or progression. These results highlight the importance of an exome-wide approach for the characterisation of patient-specific ctDNA mutation profiles prior to downstream analysis of target variants of interest, using resequencing methods [44–46]. Moreover, longitudinal tracking of patient-specific ctDNA variants revealed significant changes in mean ctDNA fractional abundances and observed clonal trends across sampled timepoints from patients, which were correlated with measurements of the tumour marker CA19-9 and/or clinical disease burden. This included one patient (patient 45) whose CA19-9 measurements were significantly below the recommended upper limit of normal (37 U/mL), indicative of a non-secretor Lewis phenotype [47]. Changes in ctDNA dynamics preceded CA19-9 levels in another (patient 13), indicating the sensitivity of ctDNA for tracking disease burden. Longitudinal evolutionary trajectories also highlighted ongoing subclonal evolution following chemotherapy treatment in these patients, demonstrating changes in the clonal architecture of ctDNA variants following treatment intervention [9, 37, 48, 49]. These results highlight the value of clonal inference and modelling for characterising longitudinal changes in ctDNA genomic profiles in PDAC and warrant further study to assess the relevance of observed clonal shifts for patient responses to treatment and overall outcomes.

Multiple trackable DDR gene variants were also identified in ctDNA through longitudinal analysis, even in cases with localised disease. Most of these variants were estimated to have a high relative clonality, further supporting their clinical potential [50]. Somatic mutations impairing the function of genes within DDR pathways can promote a defective DNA damage response in tumours, particularly in response to intra-strand crosslinks, or single-strand breaks leading to stalled replication forks and double-strand breaks induced by platinum chemotherapies and PARP inhibitors [51]. In two resectable cases sampled for this study, enrichments for the DSBR mutational signature, previously reported to be a hallmark of unstable tumour genomes in PDAC [26], were observed alongside DDR gene variants in tumour and plasma, despite the absence of *BRCA* gene mutations in one patient. Importantly, recent studies have broadened the concept of ‘BRCAness’ in PDAC, showing that cases of BRCA-deficiency are not always synonymous with BRCA-mutant tumours, providing an important putative biomarker for molecularly-guided treatment [26]. The identification of such targetable tumour alterations in ctDNA demonstrates the benefits of patient-specific ctDNA analysis to broaden existing characterisations of actionable tumour genomes in PDAC, with the potential to address the clinical imperative for targeted treatment strategies informed by tumour molecular profiles, as implemented for other solid tumour types [52].

We acknowledge that this study has several limitations. Despite the extensive longitudinal characterisations performed, exome analysis was limited to an exploratory cohort of 7 PDAC patients. Longitudinal ctDNA monitoring has not been performed extensively in PDAC cases, owing to the short patient survival times and difficulties in maintaining regular serial blood sample collections outside of an established clinical trial setting. Secondly, tumour biopsies could not be obtained from unresectable patients, as core/fine needle biopsies are not part of standard clinical care. Tumour sequencing in one resectable patient (patient 28) also failed quality control and could not be analysed. These limitations reflect challenges faced in the acquisition of suitable tissues for sequencing in the majority of PDAC patients. Most biopsies also have insufficient tumour cellularity for sequencing, which has presented a significant barrier to molecular profiling of advanced disease [14, 53]. Our results show that exome-wide ctDNA analysis can improve the molecular characterisation of both localised and advanced disease in PDAC, with the potential to circumvent the limitations of tissue-based tumour sequencing. Whilst these findings represent an important advance for tumour profiling and monitoring in the large unresectable majority of patients, we highlight that extensive comparisons between ctDNA mutation profiles and those of available paired primary and/or metastatic tumour biopsies from larger retrospectively sampled cohorts are still important to evaluate the accuracy of analytical platforms, especially for variants with allelic fractions close to the limit of detection, prior to the prospective application of exome-wide ctDNA analysis in unresectable PDAC cases.

## Conclusions

In conclusion, these findings demonstrate biological and potential clinical value for the detection and tracking of patient-specific variants in ctDNA for tumour monitoring in PDAC. We have leveraged genomic information from multiple analytical modalities, using a combination of high depth exome sequencing and serial sampling, to reliably evaluate disease burden through ctDNA and track longitudinal changes in ctDNA mutation profiles. Our results have shown that even at 1000 × depths, variants can be confidently called in ctDNA, at < 1% VAFs, which broadens the potential utility of WES for longitudinal tracking of low frequency PDAC ctDNA variants. These findings demonstrate that broad genomic profiling can enable comprehensive characterisation of tumour-associated mutations through ctDNA, leading to the identification of important molecular features with clinical implications for prognosis, monitoring and predicting treatment response in patients. Such insights would not have been possible solely through targeted sequencing of frequently mutated driver genes. These results support further investigation of personalised ctDNA monitoring as an ancillary tool to provide insight into new opportunities for molecularly defined treatment and clinical management strategies within subgroups of PDAC patients.

## Supplementary Information

**Additional file 1:** **Supplementary figure 1.** Isolated yields of cfDNA at baseline (pre-treatment) sampling in PDAC and control groups are shown in (**A**)**.** Mann-Whitney U tests were performed for comparison (*P < 0.05). Yields of overall cfDNA in PDAC cases ranged from 12.34ng/mL to 840ng/mL plasma at P1 sampling. Extracted cfDNA yields from baseline (P1) and subsequent follow-up samples (P2-P5) from sequenced PDAC cases are shown in (**B**)**.** Scatterplots showing the distribution of variant allele fractions (VAFs) of combined plasma mutations in patients with matched tissue samples available (patient 45 (left) and 95 (right)), alongside the total number of supporting reads at each variant locus, are displayed in (**C**) and (**D**)**.** Mutations specific to plasma are plotted in orange and overlapping variants shared between tumour and plasma from each patient are shown in green. Bar plots showing the distribution of the number of altered reads for somatic plasma mutation calls in each patient are shown in (**E-H**)**.** Overlapping variants shared between matched tumour and plasma from each patient are presented in (**E**) and (**G**)**.** Plasma-specific mutations are presented in (**F**) and (**H**)**.**Fragmentation profiles of plasma sequencing reads from all *n*=20 samples in our cohort containing mutant (purple) and wild-type (green) alleles at target loci for candidate tumour mutations, as identified using our pipeline, are shown in (**I**)**.** A vertical red line indicating the modal 167bp mononucleosomal fragment size is shown on the graph. **Supplementary figure 2.** Summary of analytical pipeline used for the processing andanalysis of plasma sequencing reads for identification of candidate ctDNA variant. **Supplementary figure 3.** Enriched gene signalling pathways (Reactome) amongst ctDNA variants from patients 28 (**A**), 13(**B**), 50 (**C**), 51 (**D**) and 04 (**E**)**.** Multiple aberrations were observed in ctDNA within signalling pathways representative of PDAC, with frequent mutations in genes associated with TGF-b, WNT, NOTCH signalling and chromatin modification. **Supplementary figure 4.** Bar plots showing the overall number of ctDNA mutations, (with known/predicted driver classifications) identified throughout serial plasma timepoints in patients with >2 plasma samples (**A-E**)**.** The number of ctDNA mutations varied significantly across sampled timepoints from individual patients. In all resectable patients (**A-C**), a reduction in the total number of ctDNA mutations was observed following surgical removal of primary tumour lesions (P1 to P2 sampling). Similarly, reductions in the number of ctDNA mutations were observed in unresectable patient 13, during the course of first-line chemotherapy treatment (P1 to P2). **Supplementary figure 5.** Comparison between the total number of altered copy number calls across sequenced samples is shown in (**A**)**.** The chi-squared test was performed for comparison (***P < 0.0001). The distribution of unique copy number events across individual chromosomes in tumour (left) and plasma (right) samples is displayed in (**B**), demonstrating differential enrichments for copy number gain (HLAMP, high-level amplification; AMP, amplification; GAIN, copy number gain) and loss (HOMD, homozygous deletion; HETD, heterozygous deletion) events. (**C**) Genome-wide copy number calls in plasma from one patient (patient 04) highlighted a gain (red) in copy number at chromosome 12p. (**D**) Further analysis of focal copy number calls indicated copy number gains at the KRAS locus, concurrent with the presence of KRAS G12D mutations. Copy number calls were determined using ichorCNA (top), Sequenza (middle) and CopywriteR (bottom). **Supplementary figure 6.** Bar plots showing the number of mutations within each substitution category that were identified in regions of kataegis across individual chromosomes, in each patient from our sequenced cohort (**A**)**.** Bar plots showing the total number of kataegis events detected using MAFtools in available tumour sequencing data from TCGA (PAAD-US) and ICGC (PACA-AU, PACA-CA) PDAC tumour cohorts are shown in (**B**)**.** Base substitution profiles of somatic mutations detected within regions of kataegis in each TCGA/ICGC cohort are displayed. (**C**)Notably, kataegis events co-localising with ERBB2 were not detected in TCGA/ICGC PDAC tumours. **Supplementary figure 7.** Examples of ctDNA genes containing driver mutations that were predicted to confer response to existing clinical/pre-clinical treatments using in silico predictive algorithms from Cancer Genome Interpreter, are shown. (**A**) The widths of gene segments correspond to the number of unique drug targets identified for ctDNA alterations detected within that gene. (**B**) Bar plot displaying enriched (COSMIC) mutational signatures across sequenced tumour and plasma samples. The contribution of each signature as a proportion of total signatures detected in each sample is shown. Overall, 9 COSMIC signature classes were resolved in this cohort, including 3 signatures with currently unknown aetiologies (Signature 23, Signature 25, Signature 28). **Supplementary figure 8.** Analysis of clonal dynamics and evolutionary trajectories in patients 28 (**A-C**), 95 (**D-F**) and 13 (**G-I**)**.** Longitudinally observed phylogenetic trees showing the predicted clonal evolutionary trajectories of individual ctDNA clones from each patient are shown in (**A**), (**D**) and (**G**). Scatterplots showing the estimated prevalence of inferred clones in ctDNA across sampled timepoints, are shown in (**B**), (**E**) and (**H**). Clonal diagrams of the tree structures from(**A**), (**D**) and (**G**) are displayed in (**C**), (**F**) and (**I**).

**Additional file 2:** **Supplementary Table 1. **Summary of the clinical characteristics of the study cohort.** Supplementary Table 2. **Summary of mean sequencing depths and the number of somatic variants called in tumour and PDAC plasma samples analysed. **Supplementary Table 3.** Summary of ctDNA variant allele fractions (VAFs) for potentially actionable DNA damage repair (DDR) mutations detected per patient and plasma timepoint for the exploratory cohort

## Data Availability

Sequence data has been deposited at the European Genome-phenome Archive (EGA), which is hosted by the EBI and the CRG, under accession number EGAD00001008593, accessible via https://ega-archive.org/datasets/. (Study ID: EGAS00001005981; Data Access Committee: EGAC00001002556.)

## Abbreviations

CfDNA: Cell-free DNA
CN: Copy number
CNV: Copy number variation
CP: Chronic pancreatitis
CtDNA: Circulating tumour DNA
DdPCR: Droplet digital PCR
DDR: DNA damage repair
DSBR: Double-strand break repair
HC: Healthy control
MMR: Mismatch repair
PARP: Polyadenosine-diphosphate-ribose polymerase
PDAC: Pancreatic ductal adenocarcinoma
POLN: Polymerase ν(nu)
SCNA: Somatic copy number alteration
