# Supplementary material for: Longitudinal profiling of circulating tumour DNA for tracking tumour dynamics in pancreatic cancer
Source: BMC Cancer. 2022 Apr 7;22:369. doi: 10.1186/s12885-022-09387-6 (PMC8991893; doi:10.1186/s12885-022-09387-6)
Supplement: Supplementary file 1 — Additional file 1: Supplementary figure 1. Isolated yields of cfDNA at baseline (pre-treatment) sampling in PDAC and control groups are shown in (A). Mann-Whitney U tests were performed for comparison (*P < 0.05). Yields of overall cfDNA in PDAC cases ranged from 12.34ng/mL to 840ng/mL plasma at P1 sampling. Extracted cfDNA yields from baseline (P1) and subsequent follow-up samples (P2-P5) from sequenced PDAC cases are shown in (B). Scatterplots showing the distribution of variant allele fractions (VAFs) of combined plasma mutations in patients with matched tissue samples available (patient 45 (left) and 95 (right)), alongside the total number of supporting reads at each variant locus, are displayed in (C) and (D). Mutations specific to plasma are plotted in orange and overlapping variants shared between tumour and plasma from each patient are shown in green. Bar plots showing the distribution of the number of altered reads for somatic plasma mutation calls in each patient are shown in (E-H). Overlapping variants shared between matched tumour and plasma from each patient are presented in (E) and (G). Plasma-specific mutations are presented in (F) and (H).Fragmentation profiles of plasma sequencing reads from all n=20 samples in our cohort containing mutant (purple) and wild-type (green) alleles at target loci for candidate tumour mutations, as identified using our pipeline, are shown in (I). A vertical red line indicating the modal 167bp mononucleosomal fragment size is shown on the graph. Supplementary figure 2. Summary of analytical pipeline used for the processing andanalysis of plasma sequencing reads for identification of candidate ctDNA variant. Supplementary figure 3. Enriched gene signalling pathways (Reactome) amongst ctDNA variants from patients 28 (A), 13(B), 50 (C), 51 (D) and 04 (E). Multiple aberrations were observed in ctDNA within signalling pathways representative of PDAC, with frequent mutations in genes associated with TGF-b, WNT, NOTCH signalling [file 12885_2022_9387_MOESM1_ESM.pdf]

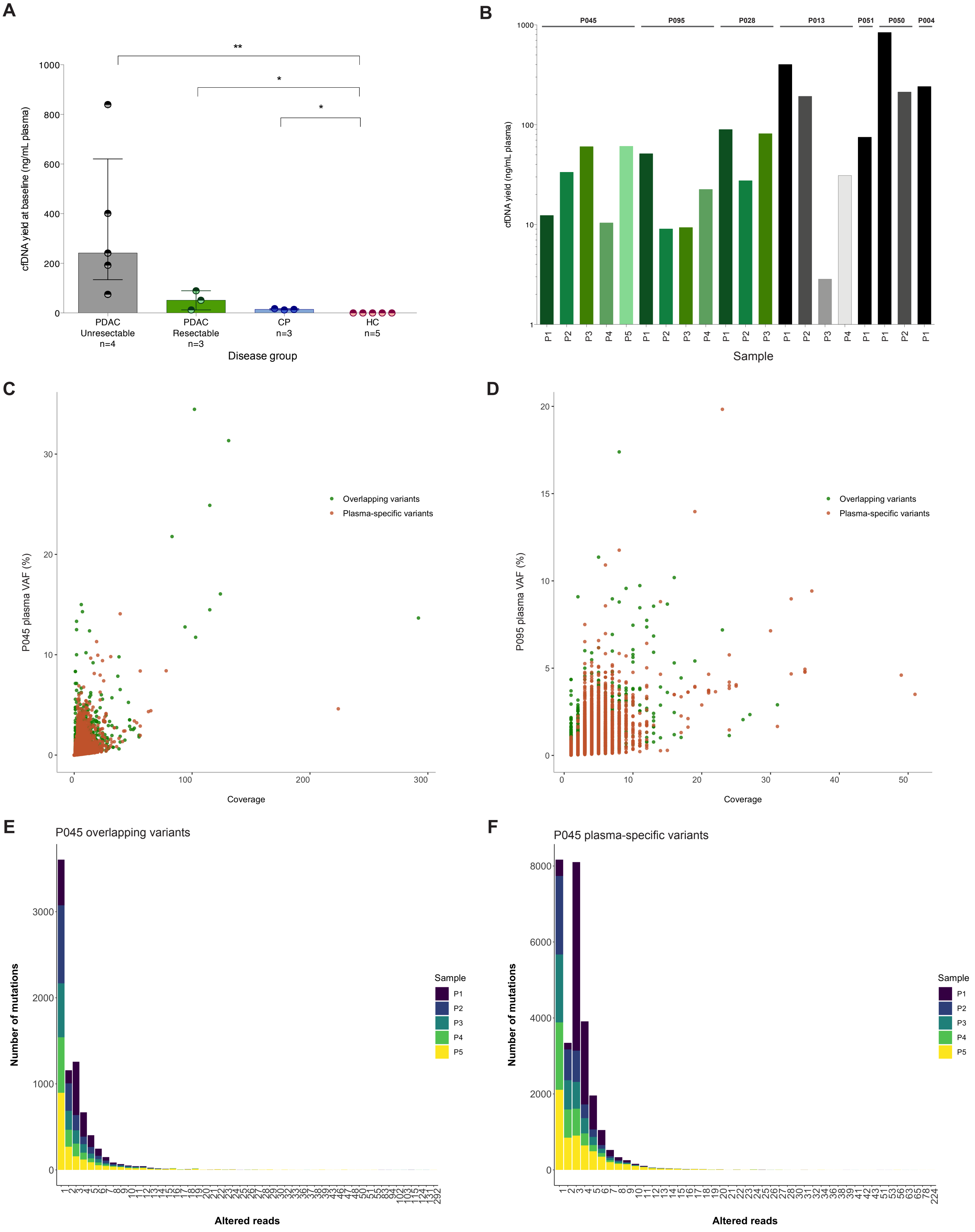
A
cfDNA yield at baseline (ng/mL plasma)
**
*
*
PDAC Unresectable n=4
PDAC Resectable n=3
CP n=3
HC n=5
Disease group
B
P045
P095
P028
P013
P051
P050
P004
cfDNA yield (ng/mL plasma)
Sample
C
P045 plasma VAF (%)
Overlapping variants
Plasma-specific variants
Coverage
D
P095 plasma VAF (%)
Overlapping variants
Plasma-specific variants
Coverage
E
P045 overlapping variants
Number of mutations
Sample
P1
P2
P3
P4
P5
Altered reads
F
P045 plasma-specific variants
Number of mutations
Sample
P1
P2
P3
P4
P5
Altered reads

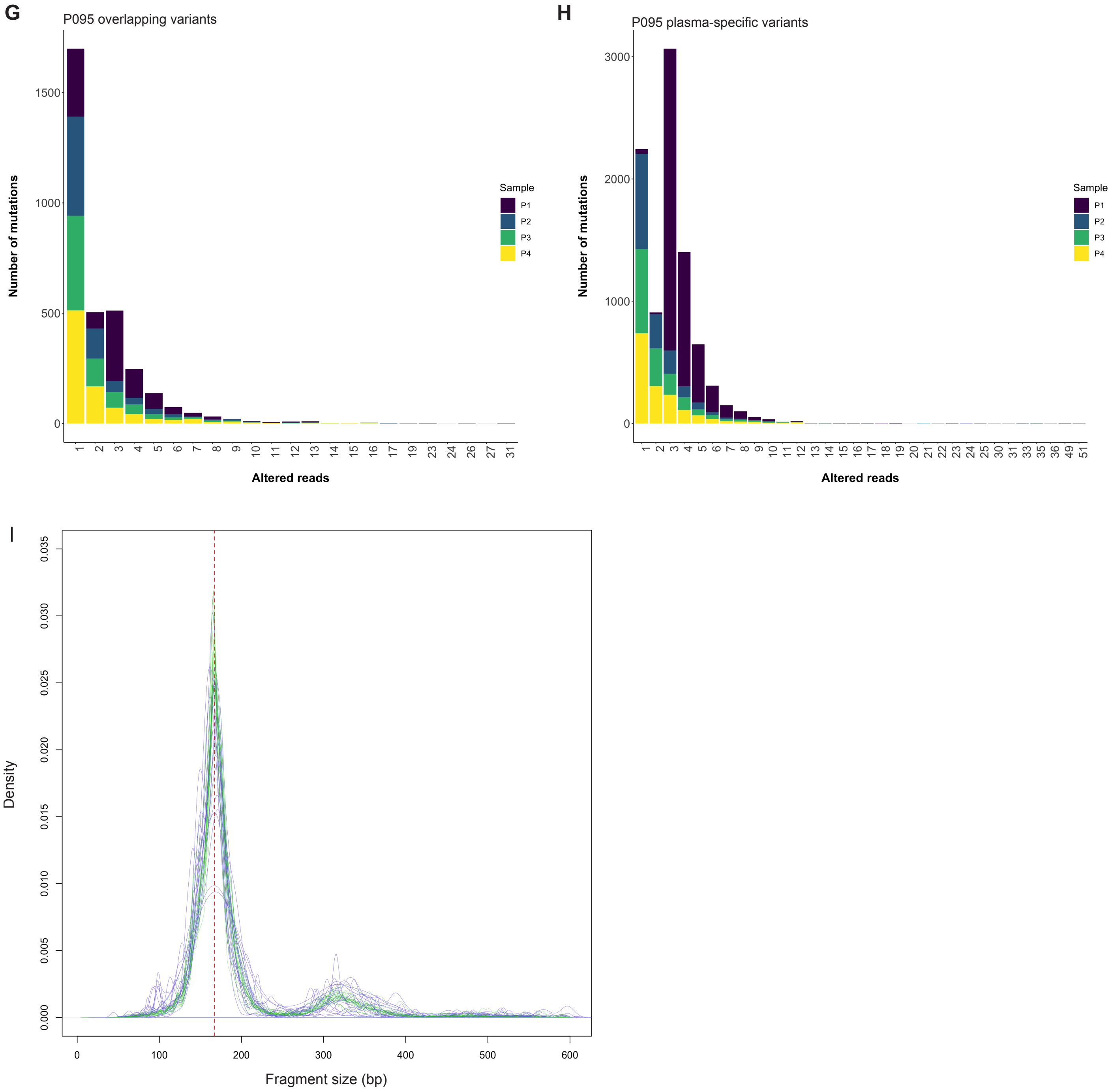

Supplementary figure 1. Isolated yields of cfDNA at baseline (pre-treatment) sampling in PDAC and control groups are shown in **(A)**. Mann-Whitney U tests were performed for comparison (*P $\leq$ 0.05). Yields of overall cfDNA in PDAC cases ranged from 12.34ng/mL to 840ng/mL plasma at P1 sampling. Extracted cfDNA yields from baseline (P1) and subsequent follow-up samples (P2-P5) from sequenced PDAC cases are shown in **(B)**. Scatterplots showing the distribution of variant allele fractions (VAFs) of combined plasma mutations in patients with matched tissue samples available (patient 45 *(left)* and 95 *(right)*), alongside the total number of supporting reads at each variant locus, are displayed in **(C)** and **(D)**. Mutations specific to plasma are plotted in *orange* and overlapping variants shared between tumour and plasma from each patient are shown in *green*. Bar plots showing the distribution of the number of altered reads for somatic plasma mutation calls in each patient are shown in **(E-H).**

Overlapping variants shared between matched tumour and plasma from each patient are presented in **(E)** and **(G).** Plasma-specific mutations are presented in **(F)** and **(H).** Fragmentation profiles of plasma sequencing reads from all n=20 samples in our cohort containing mutant *(purple)* and wild-type *(green)* alleles at target loci for candidate tumour mutations, as identified using our pipeline, are shown in **(I)**. A vertical *red* line indicating the modal 167bp mononucleosomal fragment size is shown on the graph.

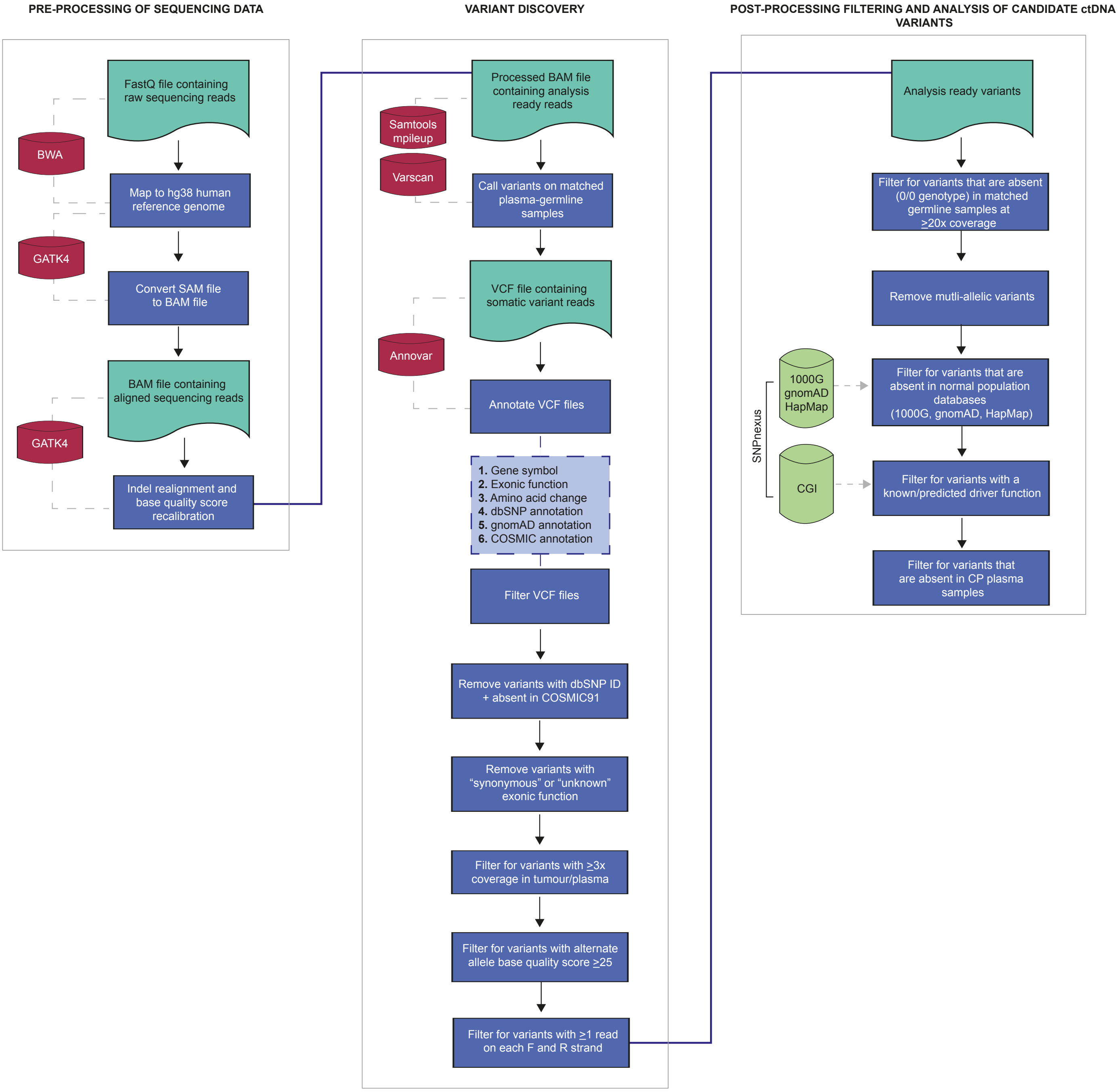

Supplementary figure 2. Summary of analytical pipeline used for the processing and analysis of plasma sequencing reads for identification of candidate ctDNA variant

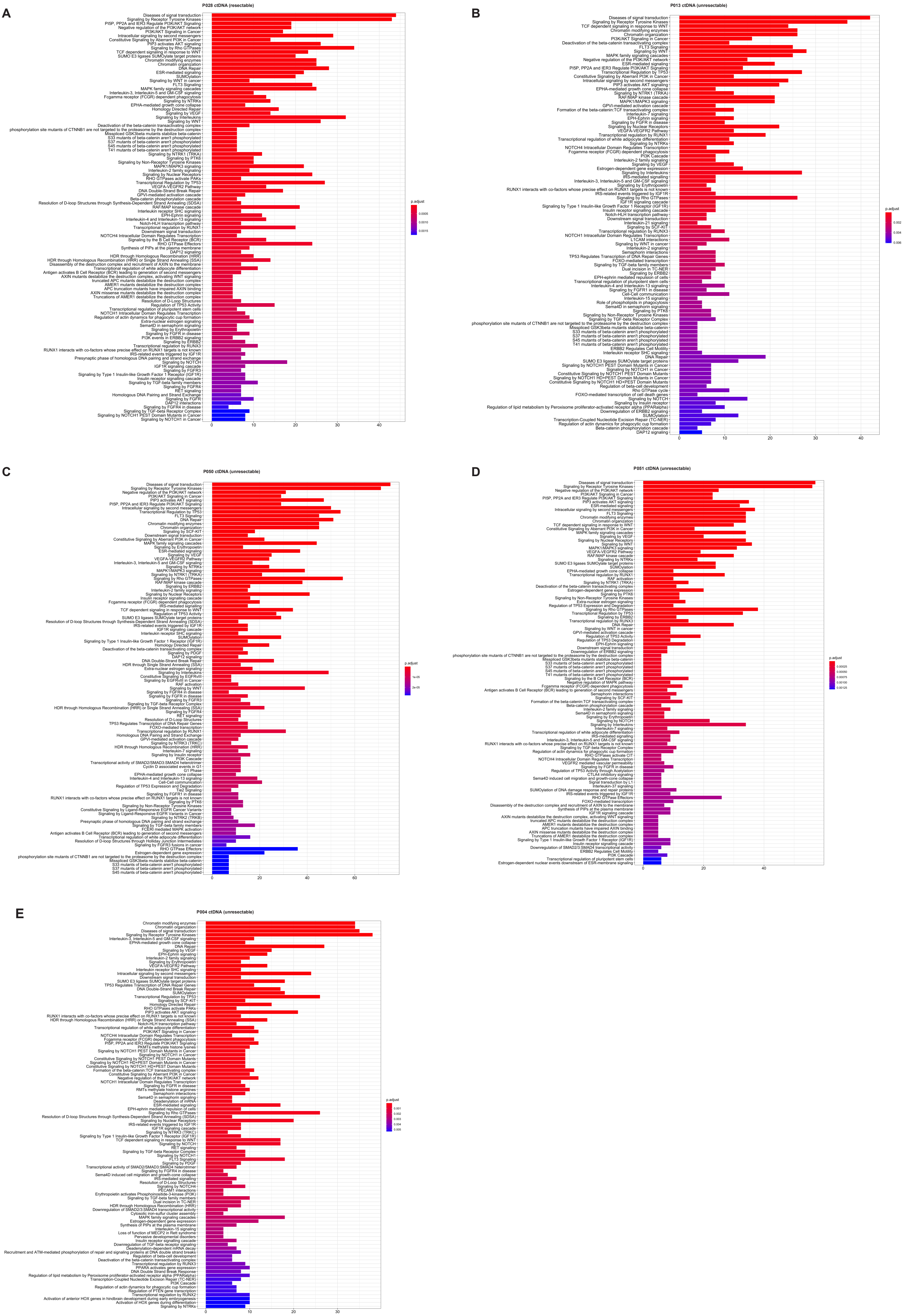
A
P028 ctDNA (resectable)
B
P013 ctDNA (unresectable)
C
P050 ctDNA (unresectable)
D
P051 ctDNA (unresectable)
E
P004 ctDNA (unresectable)
p.adjust

Supplementary figure 3. Enriched gene signalling pathways *(Reactome)* amongst ctDNA variants from patients 28 **(A),** 13 **(B),** 50 **(C),** 51 **(D)** and 04 **(E).** Multiple aberrations were observed in ctDNA within signalling pathways representative of PDAC, with frequent mutations in genes associated with TGF-β, WNT, NOTCH signalling and chromatin modification.

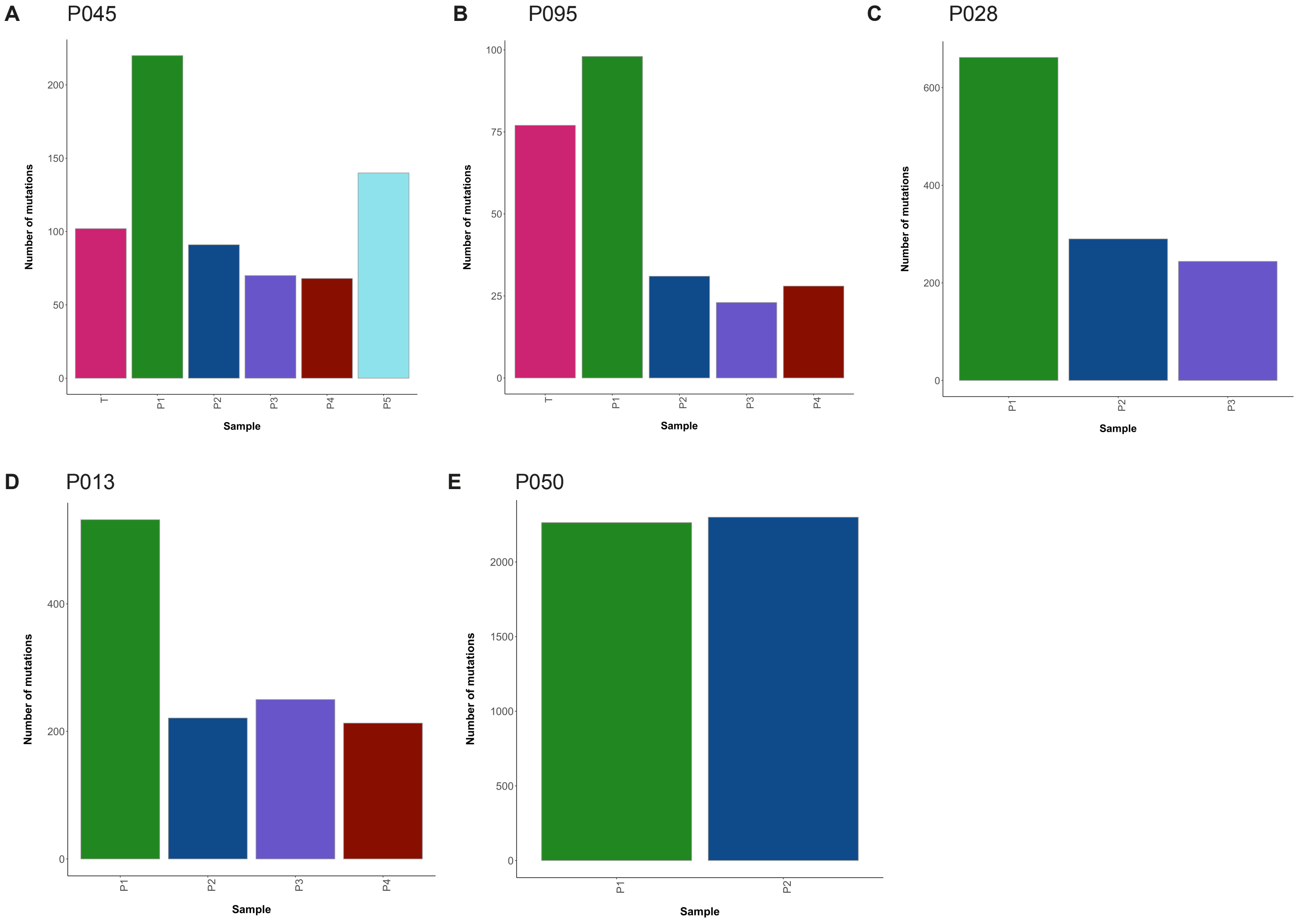

Supplementary figure 4. Bar plots showing the overall number of ctDNA mutations, (with known/predicted driver classifications) identified throughout serial plasma timepoints in patients with ≥2 plasma samples **(A-E)**. The number of ctDNA mutations varied significantly across sampled timepoints from individual patients. In all resectable patients **(A-C)**, a reduction in the total number of ctDNA mutations was observed following surgical removal of primary tumour lesions (P1 to P2 sampling). Similarly, reductions in the number of ctDNA mutations were observed in unresectable patient 13, during the course of first-line chemotherapy treatment (P1 to P2).

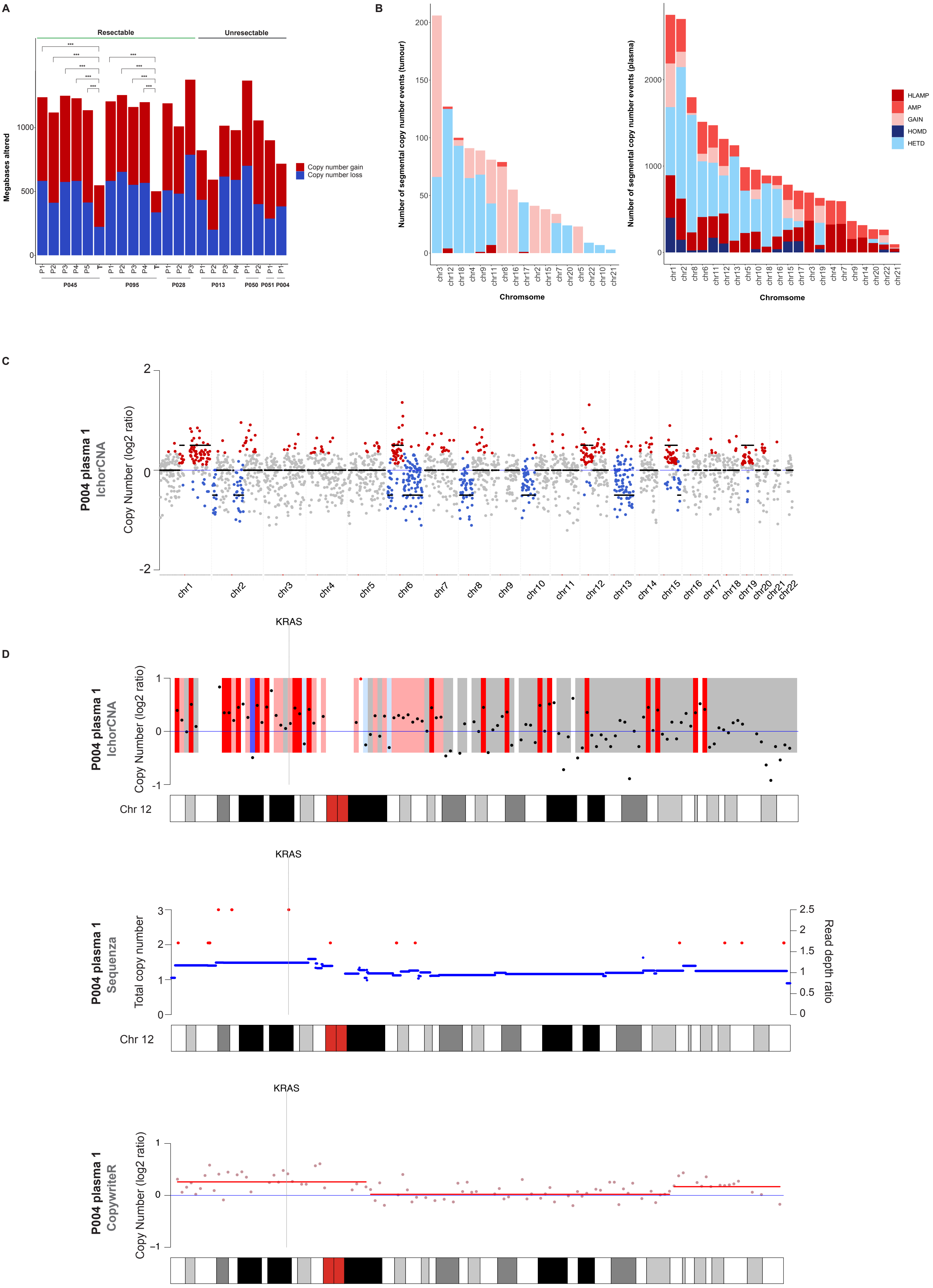
A
Resectable
Unresectable
Megabases altered
Copy number gain
Copy number loss
P045
P095
P028
P013
P050
P051
P004
B
Number of segmental copy number events (tumour)
Number of segmental copy number events (plasma)
Chromsome
HLAMP
AMP
GAIN
HOMD
HETD
C
P004 plasma 1
IchorCNA
Copy Number (log2 ratio)
D
KRAS
Chr 12
P004 plasma 1
Sequenza
Total copy number
Read depth ratio
P004 plasma 1
CopywriteR

Supplementary figure 5. Comparison between the total number of altered copy number calls across sequenced samples is shown in **(A)**. The chi-squared test was performed for comparison (***P < 0.0001). The distribution of unique copy number events across individual chromosomes in tumour *(left)* and plasma *(right)* samples is displayed in **(B)**, demonstrating differential enrichments for copy number gain (HLAMP, high-level amplification; AMP, amplification; GAIN, copy number gain) and loss (HOMD, homozygous deletion; HETD, heterozygous deletion) events. **(C)** Genome-wide copy number calls in plasma from one patient (patient 04) highlighted a gain *(red)* in copy number at chromosome 12p. **(D)** Further analysis of focal copy number calls indicated copy number gains at the *KRAS* locus, concurrent with the presence of *KRAS* G12D mutations. Copy number calls were determined using ichorCNA *(top)*, Sequenza *(middle)* and CopywriteR (bottom).

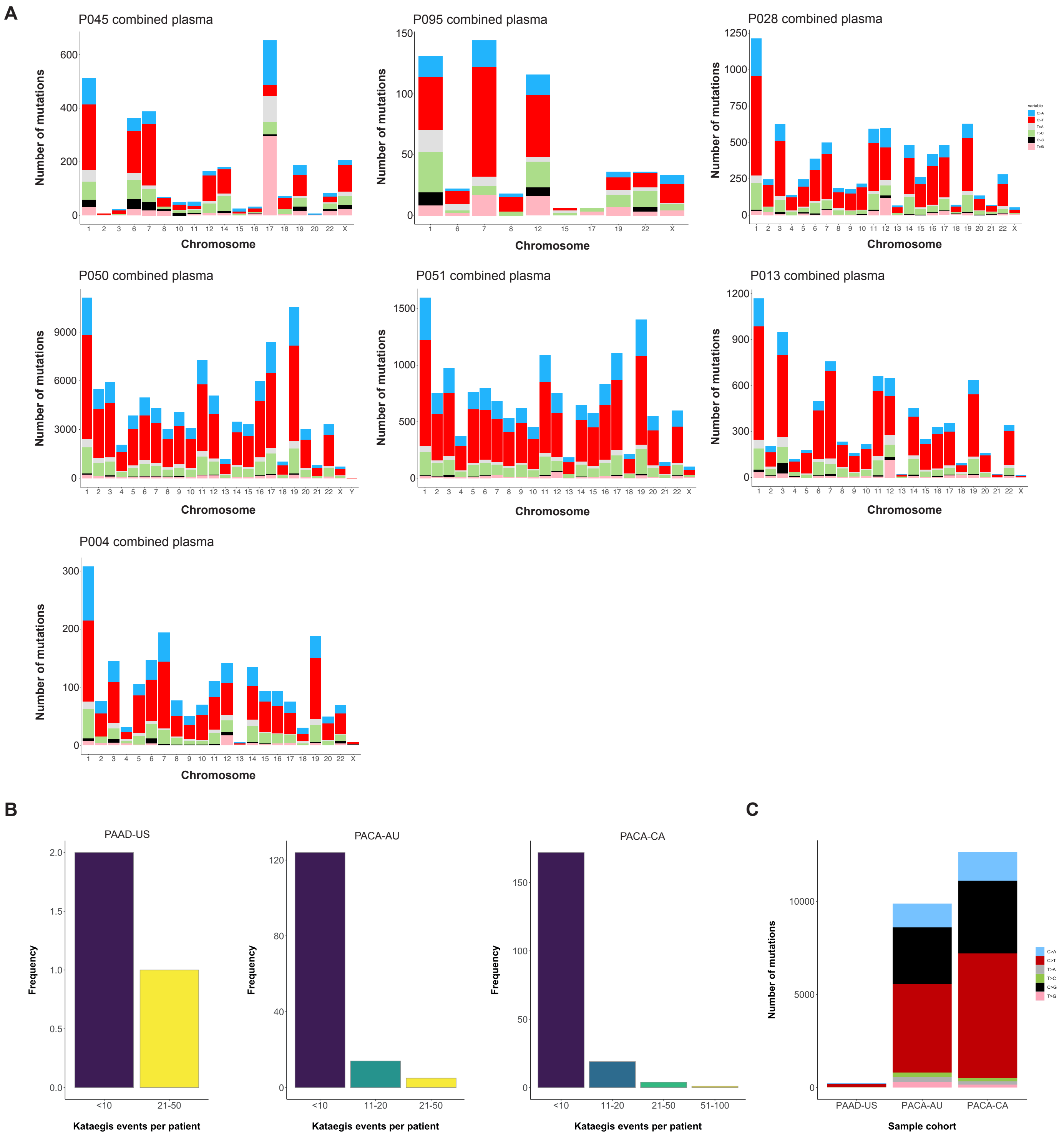

Supplementary figure 6. Bar plots showing the number of mutations within each substitution category that were identified in regions of kataegis across individual chromosomes, in each patient from our sequenced cohort **(A)**. Bar plots showing the total number of kataegis events detected using MAFtools in available tumour sequencing data from TCGA (PAAD-US) and ICGC (PACA-AU, PACA-CA) PDAC tumour cohorts are shown in **(B)**. Base substitution profiles of somatic mutations detected within regions of kataegis in each TCGA/ICGC cohort are displayed. **(C)**

Notably, kataegis events co-localising with *ERBB2* were not detected in TCGA/ICGC PDAC tumours.

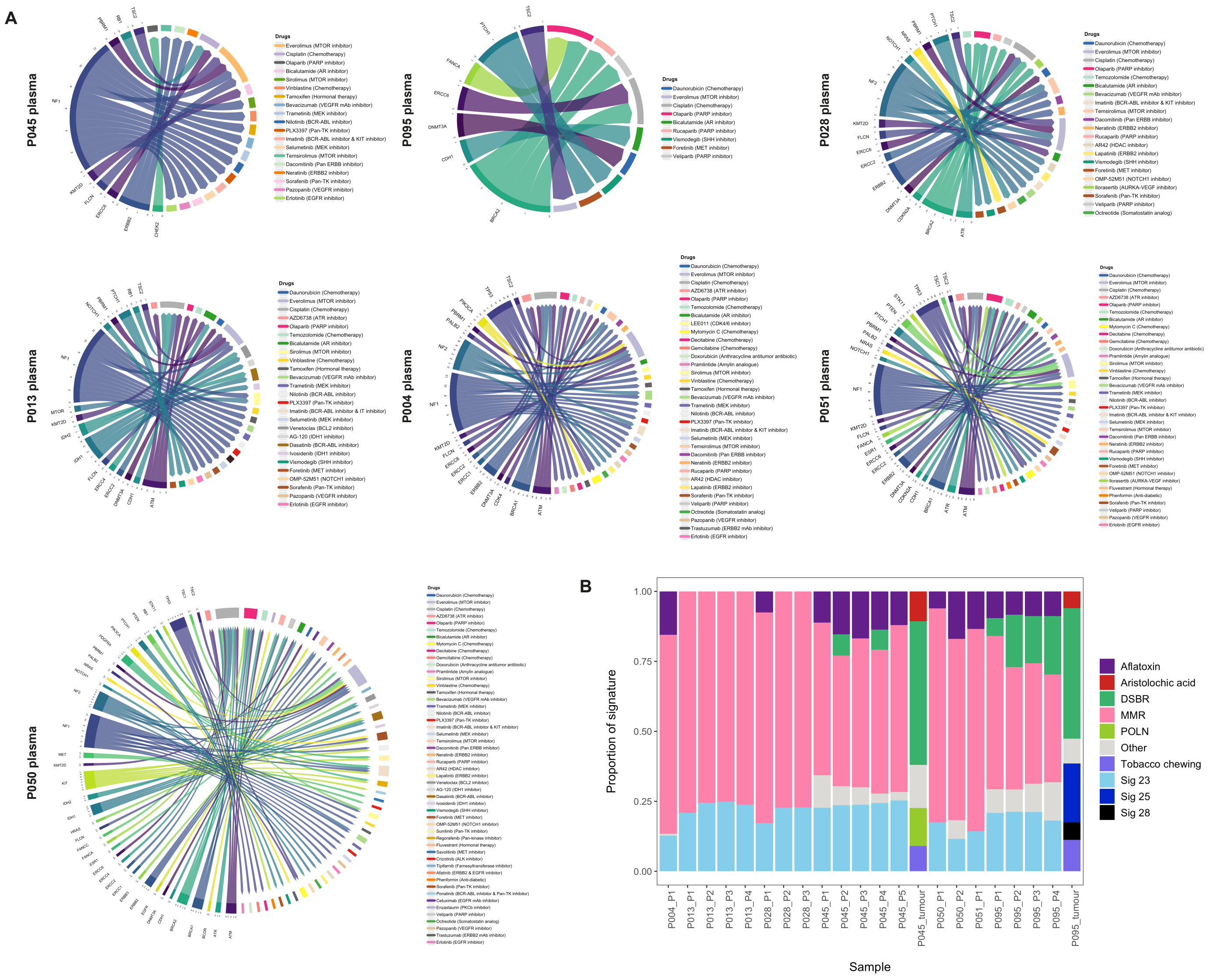

Supplementary figure 7. Examples of ctDNA genes containing driver mutations that were predicted to confer response to existing clinical/pre-clinical treatments using *in silico* predictive algorithms from *Cancer Genome Interpreter,* are shown. **(A)** The *widths* of gene segments correspond to the number of unique drug targets identified for ctDNA alterations detected within that gene. **(B)** Bar plot displaying enriched (COSMIC) mutational signatures across sequenced tumour and plasma samples. The contribution of each signature as a proportion of total signatures detected in each sample is shown. Overall, 9 COSMIC signature classes were resolved in this cohort, including 3 signatures with currently unknown aetiologies *(Signature 23, Signature 25, Signature 28)*.

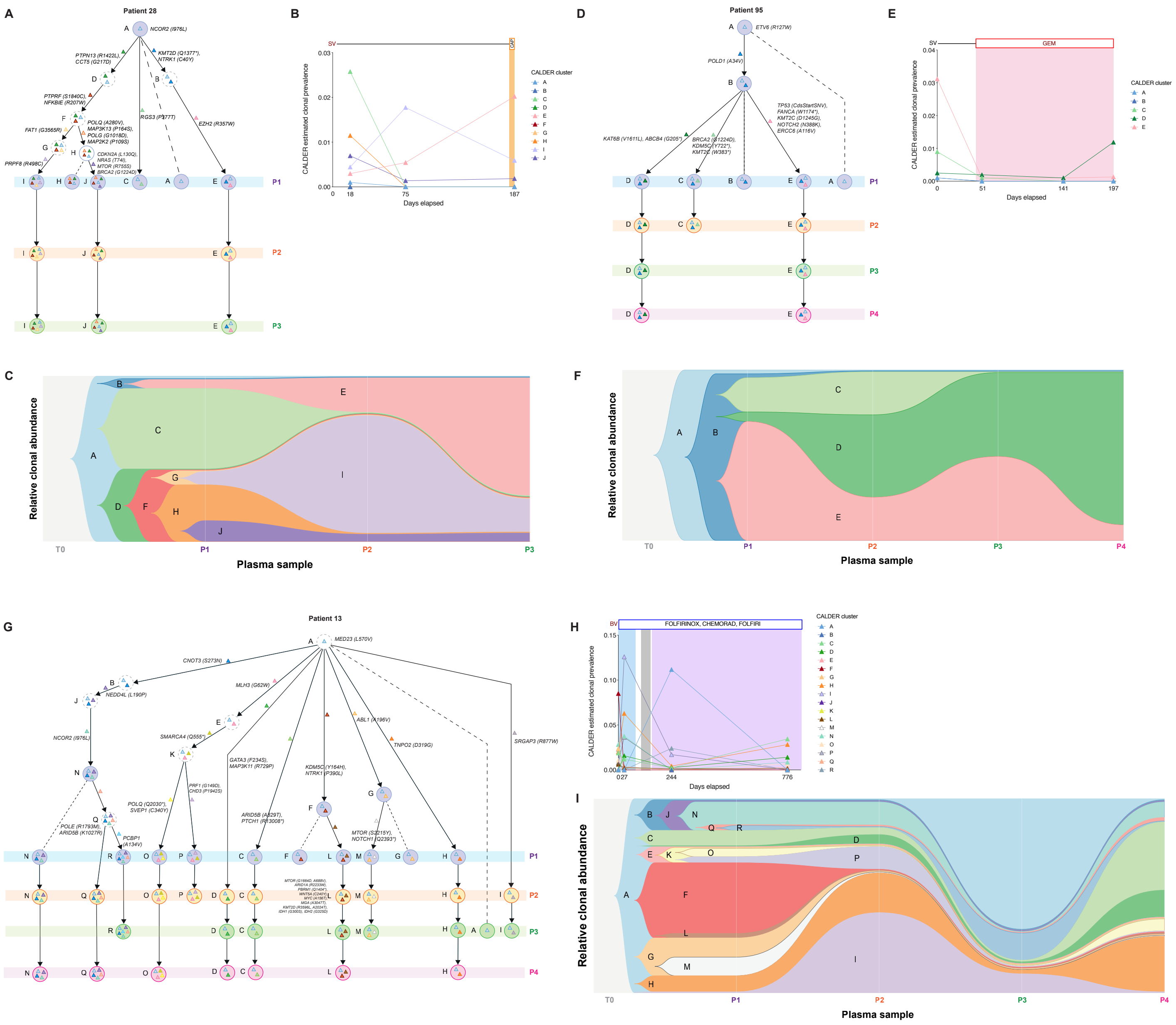

Supplementary figure 8. Analysis of clonal dynamics and evolutionary trajectories in patients 28 **(A-C)**, 95 **(D-F)** and 13 **(G-I)**. Longitudinally observed phylogenetic trees showing the predicted clonal evolutionary trajectories of individual ctDNA clones from each patient are shown in **(A)**, **(D)** and **(G)**. Scatterplots showing the estimated prevalence of inferred clones in ctDNA across sampled timepoints, are shown in **(B)**, **(E)** and **(H)**. Clonal diagrams of the tree structures from (A), (D) and (G) are displayed in **(C)**, **(F)** and **(I)**.
