# Supplementary material for: Longitudinal profiling of circulating tumour DNA for tracking tumour dynamics in pancreatic cancer
Source: BMC Cancer. 2022 Apr 7;22:369. doi: 10.1186/s12885-022-09387-6 (PMC8991893; doi:10.1186/s12885-022-09387-6)
Supplement: Supplementary file 2 — Additional file 2: Supplementary Table 1. Summary of the clinical characteristics of the study cohort. Supplementary Table 2. Summary of mean sequencing depths and the number of somatic variants called in tumour and PDAC plasma samples analysed. Supplementary Table 3. Summary of ctDNA variant allele fractions (VAFs) for potentially actionable DNA damage repair (DDR) mutations detected per patient and plasma timepoint for the exploratory cohort [file 12885_2022_9387_MOESM2_ESM.docx]

**Supplementary Table 1. Summary of the clinical characteristics of the study cohort**

| Patient ID | Sex | Ethnicity | Age at Diagnosis | Tumour location | Tumour grade | TNM at diagnosis | Site of metastases at diagnosis | Survival status | Recurrent/progressive disease |
| --- | --- | --- | --- | --- | --- | --- | --- | --- | --- |
| P045 | Female | Caucasian: Western European | 51 | Head of pancreas | Poorly differentiated | pT3N0M0 | None | Alive | No recorded recurrence |
| P095 | Female | Caucasian: Western European | 75 | Head of pancreas | Moderately differentiated | pT1N1M0 | None | Deceased | Hepatic metastases detected 6 months after baseline visit |
| P028 | Male | Caucasian: Western European | 63 | Head of pancreas | Moderately differentiated | pT2N1M0 | None | Alive | No recorded recurrence |
| P050 | Male | Caucasian: Western European | 71 | Head of pancreas | Moderately differentiated | T4N1M0 | None | Deceased | Increase in primary tumour volume observed 2 months after baseline visit |
| P013 | Male | Caucasian: Western European | 57 | Head of pancreas | Poorly differentiated | T4N0M0 | None | Alive | No recorded progression |
| P051 | Male | Afro-Caribbean | 52 | Head of pancreas | Poorly differentiated | T4N0M0 | None | Deceased | Hepatic metastasis 14 months after diagnosis |
| P004 | Male | Afro-Caribbean | 51 | Pancreatic tail | Poorly differentiated | T3N1M1 | Liver | Deceased | Progression soon after diagnosis- |

**Supplementary Table 2. Summary of mean sequencing depths and the number of somatic variants called in tumour and PDAC plasma samples analysed**

| Patient | Sample | Mean sequencing  coverage | Total number of somatic variants called | Number of filtered variants |
| --- | --- | --- | --- | --- |
| P045 | P1 | 633.164 | 11293 | 220 |
| P045 | P2 | 550.702 | 6185 | 91 |
| P045 | P3 | 637.562 | 5435 | 70 |
| P045 | P4 | 601.129 | 5113 | 68 |
| P045 | P5 | 1203.68 | 8200 | 140 |
| P045 | **T** | **109.80** | **4437** | **102** |
| P095 | P1 | 423.683 | 5511 | 98 |
| P095 | P2 | 535.135 | 2184 | 31 |
| P095 | P3 | 601.56 | 2159 | 23 |
| P095 | P4 | 649.589 | 2468 | 28 |
| P095 | **T** | **115.98** | **3217** | **77** |
| P028 | P1 | 834.838 | 36912 | 662 |
| P028 | P2 | 997.967 | 19158 | 290 |
| P028 | P3 | 816.181 | 17327 | 244 |
| P004 | P1 | 784.656 | 25036 | 474 |
| P013 | P1 | 722.893 | 31220 | 532 |
| P013 | P2 | 678.375 | 15091 | 221 |
| P013 | P3 | 910.6 | 17271 | 250 |
| P013 | P4 | 632.261 | 15524 | 213 |
| P050 | P1 | 1133.84 | 111276 | 2264 |
| P050 | P2 | 841.708 | 109093 | 2299 |
| P051 | P1 | 974.343 | 57961 | 1120 |

**Supplementary Table 3. Summary of ctDNA variant allele fractions (VAFs) for potentially actionable DNA damage repair (DDR) mutations detected per patient and plasma timepoint for the exploratory cohort**

| Sample | Chromosome | Position | Gene | Reference allele | Altered allele | VAF (%) | Variant consequence |
| --- | --- | --- | --- | --- | --- | --- | --- |
| P013_P1 | chr2 | 127286988 | ERCC3 | C | T | 0.37 | Missense |
| P013_P1 | chr3 | 10035232 | FANCD2 | A | G | 0.82 | Missense |
| P013_P1 | chr3 | 10078096 | FANCD2 | C | T | 2.08 | Nonsense |
| P013_P1 | chr3 | 121460067 | POLQ | T | C | 1.17 | Missense |
| P013_P1 | chr3 | 121481695 | POLQ | G | A | 1.15 | Nonsense |
| P013_P1 | chr3 | 136502668 | STAG1 | G | A | 0.89 | Missense |
| P013_P1 | chr4 | 57022241 | POLR2B | G | A | 0.79 | Missense |
| P013_P1 | chr4 | 57025536 | POLR2B | C | T | 0.64 | Missense |
| P013_P1 | chr6 | 35462802 | FANCE | C | A | 0.29 | Missense |
| P013_P1 | chr9 | 130862800 | ABL1 | C | T | 3.17 | Missense |
| P013_P1 | chr9 | 130872154 | ABL1 | T | C | 0.43 | Missense |
| P013_P1 | chr9 | 130872866 | ABL1 | G | A | 1.5 | Missense |
| P013_P1 | chr9 | 130885346 | ABL1 | C | A | 0.15 | Missense |
| P013_P1 | chr11 | 3676259 | NUP98 | G | A | 0.88 | Missense |
| P013_P1 | chr11 | 3723286 | NUP98 | C | T | 0.31 | Missense |
| P013_P1 | chr11 | 108325410 | ATM | G | A | 0.39 | Missense |
| P013_P1 | chr11 | 108353844 | ATM | G | A | 0.36 | Missense |
| P013_P1 | chr12 | 132624976 | POLE | C | A | 0.69 | Missense |
| P013_P1 | chr12 | 132641647 | POLE | C | A | 0.39 | Missense |
| P013_P1 | chr13 | 48362928 | RB1 | T | C | 0.47 | Missense |
| P013_P1 | chr15 | 28146243 | HERC2 | G | A | 0.34 | Missense |
| P013_P1 | chr15 | 28265861 | HERC2 | T | C | 0.5 | Missense |
| P013_P1 | chr15 | 34910231 | AQR | C | T | 0.47 | Missense |
| P013_P1 | chr15 | 34969547 | AQR | C | T | 0.48 | Missense |
| P013_P1 | chr15 | 40728761 | RAD51 | C | A | 0.35 | Missense |
| P013_P1 | chr15 | 43409023 | TP53BP1 | G | A | 0.62 | Missense |
| P013_P1 | chr15 | 43415780 | TP53BP1 | G | A | 0.4 | Missense |
| P013_P1 | chr15 | 43475628 | TP53BP1 | G | A | 0.95 | Missense |
| P013_P1 | chr15 | 89258756 | FANCI | T | C | 0.71 | Missense |
| P013_P1 | chr16 | 2043612 | NTHL1 | C | T | 1.25 | Missense |
| P013_P1 | chr16 | 2046166 | NTHL1 | C | A | 0.4 | Missense |
| P013_P1 | chr16 | 56841735 | NUP93 | G | A | 0.31 | Missense |
| P013_P1 | chr17 | 35101340 | RAD51D | C | A | 0.34 | Missense |
| P013_P1 | chr17 | 39531020 | CDK12 | G | T | 0.24 | Missense |
| P013_P1 | chr19 | 45352799 | ERCC2 | C | T | 0.15 | Missense |
| P013_P1 | chr19 | 45353134 | ERCC2 | C | T | 0.44 | Missense |
| P013_P1 | chr19 | 45355694 | ERCC2 | C | T | 0.54 | Missense |
| P013_P1 | chr19 | 50402288 | POLD1 | C | A | 0.22 | Missense |
| P013_P1 | chr22 | 41176407 | EP300 | C | T | 0.66 | Missense |
| P013_P1 | chr22 | 41178813 | EP300 | G | T | 0.36 | Missense |
| P013_P1 | chr9 | 130854954 | ABL1 | C | A | 0.19 | Missense |
| P013_P2 | chr9 | 130872866 | ABL1 | G | A | 0.83 | Missense |
| P013_P2 | chr11 | 108325410 | ATM | G | A | 0.16 | Missense |
| P013_P2 | chr11 | 108353844 | ATM | G | A | 0.12 | Missense |
| P013_P2 | chr15 | 28146243 | HERC2 | G | A | 0.13 | Missense |
| P013_P2 | chr15 | 28265861 | HERC2 | T | C | 0.52 | Missense |
| P013_P2 | chr15 | 34910231 | AQR | C | T | 0.15 | Missense |
| P013_P2 | chr15 | 43475628 | TP53BP1 | G | A | 1.66 | Missense |
| P013_P2 | chr16 | 2043612 | NTHL1 | C | T | 0.35 | Missense |
| P013_P2 | chr17 | 35101340 | RAD51D | C | A | 0.11 | Missense |
| P013_P2 | chr17 | 39531020 | CDK12 | G | T | 0.24 | Missense |
| P013_P2 | chr19 | 45355694 | ERCC2 | C | T | 0.24 | Missense |
| P013_P2 | chr19 | 50402288 | POLD1 | C | A | 0.3 | Missense |
| P013_P2 | chr3 | 121493544 | POLQ | G | A | 0.35 | Missense |
| P013_P2 | chr9 | 130854954 | ABL1 | C | A | 0.24 | Missense |
| P013_P2 | chr16 | 13947797 | ERCC4 | G | A | 0.22 | Missense |
| P013_P2 | chr22 | 41150118 | EP300 | C | T | 0.5 | Nonsense |
| P013_P3 | chr2 | 127286988 | ERCC3 | C | T | 0.11 | Missense |
| P013_P3 | chr3 | 10078096 | FANCD2 | C | T | 0.63 | Nonsense |
| P013_P3 | chr9 | 130872154 | ABL1 | T | C | 0.12 | Missense |
| P013_P3 | chr9 | 130872866 | ABL1 | G | A | 0.08 | Missense |
| P013_P3 | chr9 | 130885346 | ABL1 | C | A | 0.04 | Missense |
| P013_P3 | chr11 | 108325410 | ATM | G | A | 0.18 | Missense |
| P013_P3 | chr11 | 108353844 | ATM | G | A | 0.41 | Missense |
| P013_P3 | chr12 | 132624976 | POLE | C | A | 0.26 | Missense |
| P013_P3 | chr12 | 132641647 | POLE | C | A | 1.55 | Missense |
| P013_P3 | chr15 | 28146243 | HERC2 | G | A | 0.15 | Missense |
| P013_P3 | chr15 | 28265861 | HERC2 | T | C | 0.64 | Missense |
| P013_P3 | chr15 | 34910231 | AQR | C | T | 0.14 | Missense |
| P013_P3 | chr15 | 34969547 | AQR | C | T | 0.12 | Missense |
| P013_P3 | chr15 | 40728761 | RAD51 | C | A | 0.09 | Missense |
| P013_P3 | chr15 | 43409023 | TP53BP1 | G | A | 0.12 | Missense |
| P013_P3 | chr15 | 43415780 | TP53BP1 | G | A | 0.41 | Missense |
| P013_P3 | chr15 | 43475628 | TP53BP1 | G | A | 0.3 | Missense |
| P013_P3 | chr17 | 35101340 | RAD51D | C | A | 0.09 | Missense |
| P013_P3 | chr19 | 45352799 | ERCC2 | C | T | 0.04 | Missense |
| P013_P3 | chr19 | 45353134 | ERCC2 | C | T | 0.15 | Missense |
| P013_P3 | chr19 | 45355694 | ERCC2 | C | T | 0.15 | Missense |
| P013_P3 | chr22 | 41176407 | EP300 | C | T | 0.13 | Missense |
| P013_P3 | chr3 | 121493544 | POLQ | G | A | 0.09 | Missense |
| P013_P3 | chr9 | 130854954 | ABL1 | C | A | 0.14 | Missense |
| P013_P3 | chr16 | 13947797 | ERCC4 | G | A | 0.28 | Missense |
| P013_P3 | chr22 | 41150118 | EP300 | C | T | 0.13 | Nonsense |
| P013_P4 | chr3 | 10035232 | FANCD2 | A | G | 0.22 | Missense |
| P013_P4 | chr3 | 121481695 | POLQ | G | A | 0.68 | Nonsense |
| P013_P4 | chr4 | 57025536 | POLR2B | C | T | 0.24 | Missense |
| P013_P4 | chr9 | 130872866 | ABL1 | G | A | 0.1 | Missense |
| P013_P4 | chr9 | 130885346 | ABL1 | C | A | 0.04 | Missense |
| P013_P4 | chr11 | 108353844 | ATM | G | A | 0.5 | Missense |
| P013_P4 | chr15 | 28146243 | HERC2 | G | A | 0.11 | Missense |
| P013_P4 | chr15 | 40728761 | RAD51 | C | A | 0.34 | Missense |
| P013_P4 | chr17 | 39531020 | CDK12 | G | T | 0.06 | Missense |
| P013_P4 | chr19 | 45352799 | ERCC2 | C | T | 0.2 | Missense |
| P013_P4 | chr19 | 45353134 | ERCC2 | C | T | 0.11 | Missense |
| P013_P4 | chr19 | 45355694 | ERCC2 | C | T | 0.09 | Missense |
| P013_P4 | chr22 | 41178813 | EP300 | G | T | 0.03 | Missense |
| P013_P4 | chr3 | 121493544 | POLQ | G | A | 0.18 | Missense |
| P013_P4 | chr9 | 130854954 | ABL1 | C | A | 0.14 | Missense |
| P013_P4 | chr16 | 13947797 | ERCC4 | G | A | 0.2 | Missense |
| P028_P1 | chr1 | 35747937 | CLSPN | T | C | 0.58 | Missense |
| P028_P1 | chr2 | 127272935 | ERCC3 | T | A | 0.39 | Missense |
| P028_P1 | chr3 | 14147303 | XPC | C | A | 0.43 | Missense |
| P028_P1 | chr3 | 14172900 | XPC | T | A | 0.41 | Missense |
| P028_P1 | chr3 | 52407402 | BAP1 | G | A | 0.67 | Missense |
| P028_P1 | chr3 | 52407418 | BAP1 | C | T | 0.5 | Missense |
| P028_P1 | chr3 | 121493494 | POLQ | C | T | 0.46 | Missense |
| P028_P1 | chr3 | 121533111 | POLQ | G | A | 1.12 | Missense |
| P028_P1 | chr3 | 142524117 | ATR | C | T | 0.33 | Missense |
| P028_P1 | chr3 | 142550230 | ATR | G | A | 0.77 | Nonsense |
| P028_P1 | chr4 | 57024011 | POLR2B | C | T | 0.82 | Missense |
| P028_P1 | chr4 | 57024917 | POLR2B | C | T | 0.96 | Missense |
| P028_P1 | chr7 | 5987328 | PMS2 | G | T | 0.16 | Missense |
| P028_P1 | chr8 | 31150411 | WRN | C | A | 0.24 | Missense |
| P028_P1 | chr8 | 116849008 | RAD21 | C | T | 0.36 | Missense |
| P028_P1 | chr9 | 21970970 | CDKN2A | A | T | 0.32 | Missense |
| P028_P1 | chr9 | 97685009 | XPA | C | A | 0.41 | Missense |
| P028_P1 | chr9 | 130854917 | ABL1 | A | G | 0.21 | Missense |
| P028_P1 | chr9 | 130854954 | ABL1 | C | T | 0.62 | Missense |
| P028_P1 | chr9 | 130878514 | ABL1 | G | A | 0.73 | Missense |
| P028_P1 | chr9 | 130880556 | ABL1 | C | A | 0.63 | Missense |
| P028_P1 | chr10 | 49470298 | ERCC6 | C | A | 0.28 | Missense |
| P028_P1 | chr10 | 129707972 | MGMT | C | T | 0.63 | Missense |
| P028_P1 | chr11 | 3702698 | NUP98 | C | A | 1.36 | Missense |
| P028_P1 | chr11 | 3713895 | NUP98 | T | C | 0.27 | Missense |
| P028_P1 | chr11 | 61795593 | FEN1 | G | T | 1.3 | Missense |
| P028_P1 | chr12 | 132673592 | POLE | C | T | 0.34 | Missense |
| P028_P1 | chr13 | 32338026 | BRCA2 | G | A | 0.35 | Missense |
| P028_P1 | chr15 | 28175613 | HERC2 | G | A | 0.3 | Missense |
| P028_P1 | chr15 | 28228250 | HERC2 | A | G | 0.92 | Missense |
| P028_P1 | chr15 | 28274321 | HERC2 | G | A | 0.43 | Missense |
| P028_P1 | chr15 | 43408981 | TP53BP1 | C | T | 0.56 | Missense |
| P028_P1 | chr15 | 89285108 | FANCI | G | T | 0.33 | Missense |
| P028_P1 | chr15 | 89300377 | FANCI | C | T | 0.27 | Nonsense |
| P028_P1 | chr15 | 90785017 | BLM | C | T | 0.41 | Missense |
| P028_P1 | chr16 | 2043689 | NTHL1 | A | G | 0.77 | Missense |
| P028_P1 | chr16 | 56831868 | NUP93 | T | C | 0.52 | Missense |
| P028_P1 | chr16 | 56831912 | NUP93 | T | C | 0.45 | Missense |
| P028_P1 | chr16 | 56834185 | NUP93 | T | C | 0.29 | Missense |
| P028_P1 | chr17 | 35107041 | RAD51D | C | T | 0.81 | Missense |
| P028_P1 | chr17 | 39471523 | CDK12 | C | T | 0.34 | Missense |
| P028_P1 | chr17 | 58696823 | RAD51C | C | A | 0.48 | Missense |
| P028_P1 | chr17 | 61808586 | BRIP1 | C | T | 0.29 | Missense |
| P028_P1 | chr19 | 45352345 | ERCC2 | G | A | 1.45 | Missense |
| P028_P1 | chr19 | 45357524 | ERCC2 | C | T | 0.49 | Missense |
| P028_P1 | chr19 | 45357542 | ERCC2 | A | G | 0.22 | Missense |
| P028_P1 | chr19 | 45364253 | ERCC2 | A | T | 0.87 | Missense |
| P028_P1 | chr19 | 50403565 | POLD1 | C | A | 0.14 | Missense |
| P028_P1 | chr19 | 50403571 | POLD1 | C | A | 0.19 | Missense |
| P028_P1 | chr20 | 63690205 | RTEL1 | C | T | 0.51 | Nonsense |
| P028_P1 | chr22 | 41117210 | EP300 | G | T | 0.88 | Nonsense |
| P028_P1 | chr22 | 41117696 | EP300 | C | T | 0.92 | Nonsense |
| P028_P1 | chr22 | 41127584 | EP300 | G | A | 0.57 | Missense |
| P028_P1 | chr22 | 41150133 | EP300 | C | T | 0.37 | Nonsense |
| P028_P1 | chr22 | 41157253 | EP300 | C | T | 0.46 | Nonsense |
| P028_P1 | chr22 | 41157280 | EP300 | T | C | 0.53 | Missense |
| P028_P1 | chr22 | 41168771 | EP300 | C | A | 0.81 | Missense |
| P028_P1 | chr22 | 41176335 | EP300 | T | A | 0.29 | Missense |
| P028_P1 | chr22 | 41176903 | EP300 | G | A | 0.35 | Missense |
| P028_P1 | chr22 | 41178279 | EP300 | C | T | 0.59 | Nonsense |
| P028_P1 | chrX | 53381087 | SMC1A | G | T | 0.74 | Missense |
| P028_P2 | chr3 | 14147303 | XPC | C | A | 0.07 | Missense |
| P028_P2 | chr3 | 121533111 | POLQ | G | A | 0.35 | Missense |
| P028_P2 | chr4 | 57024917 | POLR2B | C | T | 0.17 | Missense |
| P028_P2 | chr8 | 31150411 | WRN | C | A | 0.09 | Missense |
| P028_P2 | chr8 | 116849008 | RAD21 | C | T | 0.3 | Missense |
| P028_P2 | chr9 | 130854954 | ABL1 | C | T | 0.41 | Missense |
| P028_P2 | chr9 | 130878514 | ABL1 | G | A | 0.18 | Missense |
| P028_P2 | chr11 | 3713895 | NUP98 | T | C | 0.07 | Missense |
| P028_P2 | chr11 | 61795593 | FEN1 | G | T | 0.17 | Missense |
| P028_P2 | chr13 | 32338026 | BRCA2 | G | A | 0.12 | Missense |
| P028_P2 | chr15 | 28175613 | HERC2 | G | A | 0.09 | Missense |
| P028_P2 | chr15 | 28228250 | HERC2 | A | G | 0.18 | Missense |
| P028_P2 | chr15 | 89300377 | FANCI | C | T | 0.18 | Nonsense |
| P028_P2 | chr15 | 90785017 | BLM | C | T | 0.33 | Missense |
| P028_P2 | chr16 | 2043689 | NTHL1 | A | G | 0.13 | Missense |
| P028_P2 | chr16 | 56834185 | NUP93 | T | C | 0.16 | Missense |
| P028_P2 | chr17 | 61808586 | BRIP1 | C | T | 0.45 | Missense |
| P028_P2 | chr19 | 45352345 | ERCC2 | G | A | 0.19 | Missense |
| P028_P2 | chr19 | 45357524 | ERCC2 | C | T | 0.43 | Missense |
| P028_P2 | chr20 | 63690205 | RTEL1 | C | T | 0.47 | Nonsense |
| P028_P2 | chr22 | 41117696 | EP300 | C | T | 0.21 | Nonsense |
| P028_P2 | chr22 | 41127584 | EP300 | G | A | 0.2 | Missense |
| P028_P2 | chr22 | 41176903 | EP300 | G | A | 0.23 | Missense |
| P028_P2 | chr22 | 41178279 | EP300 | C | T | 0.06 | Nonsense |
| P028_P3 | chr3 | 14147303 | XPC | C | A | 0.08 | Missense |
| P028_P3 | chr3 | 121533111 | POLQ | G | A | 0.38 | Missense |
| P028_P3 | chr7 | 5987328 | PMS2 | G | T | 0.16 | Missense |
| P028_P3 | chr8 | 116849008 | RAD21 | C | T | 0.09 | Missense |
| P028_P3 | chr9 | 130854917 | ABL1 | A | G | 0.07 | Missense |
| P028_P3 | chr9 | 130854954 | ABL1 | C | T | 0.08 | Missense |
| P028_P3 | chr9 | 130878514 | ABL1 | G | A | 0.12 | Missense |
| P028_P3 | chr10 | 49470298 | ERCC6 | C | A | 0.19 | Missense |
| P028_P3 | chr12 | 132673592 | POLE | C | T | 0.12 | Missense |
| P028_P3 | chr13 | 32338026 | BRCA2 | G | A | 0.45 | Missense |
| P028_P3 | chr15 | 28175613 | HERC2 | G | A | 0.07 | Missense |
| P028_P3 | chr15 | 89300377 | FANCI | C | T | 0.62 | Nonsense |
| P028_P3 | chr17 | 39471523 | CDK12 | C | T | 0.06 | Missense |
| P028_P3 | chr17 | 58696823 | RAD51C | C | A | 0.6 | Missense |
| P028_P3 | chr19 | 45352345 | ERCC2 | G | A | 0.21 | Missense |
| P028_P3 | chr19 | 45357524 | ERCC2 | C | T | 0.21 | Missense |
| P028_P3 | chr19 | 45364253 | ERCC2 | A | T | 0.24 | Missense |
| P028_P3 | chr20 | 63690205 | RTEL1 | C | T | 0.17 | Nonsense |
| P028_P3 | chr22 | 41117696 | EP300 | C | T | 0.21 | Nonsense |
| P028_P3 | chr22 | 41127584 | EP300 | G | A | 0.36 | Missense |
| P028_P3 | chr22 | 41176903 | EP300 | G | A | 0.17 | Missense |
| P045_P1 | chr2 | 47790958 | MSH6 | G | A | 0.99 | Missense |
| P045_P1 | chr3 | 121539469 | POLQ | T | C | 2.06 | Missense |
| P045_P1 | chr7 | 5529185 | ACTB | CT | AT | 0.95 | Missense |
| P045_P1 | chr9 | 130862799 | ABL1 | G | A | 0.44 | Missense |
| P045_P1 | chr10 | 49524753 | ERCC6 | C | T | 0.85 | Missense |
| P045_P1 | chr12 | 132659494 | POLE | C | A | 0.47 | Missense |
| P045_P1 | chr12 | 132661157 | POLE | C | A | 2.22 | Missense |
| P045_P1 | chr13 | 48465206 | RB1 | C | T | 0.69 | Missense |
| P045_P1 | chr15 | 28117138 | HERC2 | C | T | 0.31 | Missense |
| P045_P1 | chr15 | 28177015 | HERC2 | A | T | 0.63 | Missense |
| P045_P1 | chr15 | 28198745 | HERC2 | G | A | 1.09 | Nonsense |
| P045_P1 | chr15 | 43447433 | TP53BP1 | C | A | 2.63 | Missense |
| P045_P1 | chr15 | 43493041 | TP53BP1 | C | T | 0.39 | CdsStartSNV |
| P045_P1 | chr15 | 90803587 | BLM | C | T | 1.29 | Missense |
| P045_P1 | chr16 | 56758587 | NUP93 | C | T | 0.49 | Nonsense |
| P045_P1 | chr17 | 39494623 | CDK12 | A | C | 0.28 | Missense |
| P045_P1 | chr19 | 50409150 | POLD1 | C | A | 0.78 | Missense |
| P045_P1 | chr19 | 50413764 | POLD1 | C | A | 0.65 | Missense |
| P045_P1 | chr22 | 41176515 | EP300 | GTACTGT | ATACTGT | 1.66 | Missense |
| P045_P2 | chr3 | 121493478 | POLQ | C | T | 0.23 | Missense |
| P045_P2 | chr13 | 48465206 | RB1 | C | T | 0.54 | Missense |
| P045_P2 | chr15 | 28198745 | HERC2 | G | A | 0.13 | Nonsense |
| P045_P2 | chr15 | 90803587 | BLM | C | T | 0.36 | Missense |
| P045_P2 | chr17 | 39501259 | CDK12 | A | C | 0.69 | Missense |
| P045_P2 | chr17 | 39501261 | CDK12 | C | T | 0.32 | Missense |
| P045_P2 | chr19 | 50413764 | POLD1 | C | A | 0.39 | Missense |
| P045_P2 | chr22 | 28734705 | CHEK2 | T | A | 0.29 | Missense |
| P045_P2 | chr22 | 41176515 | EP300 | GTACTGT | ATACTGT | 0.92 | Missense |
| P045_P3 | chr2 | 47799146 | MSH6 | A | T | 0.39 | Missense |
| P045_P3 | chr7 | 5529185 | ACTB | CT | AT | 0.5 | Missense |
| P045_P3 | chr12 | 132659494 | POLE | C | A | 0.19 | Missense |
| P045_P3 | chr15 | 28198745 | HERC2 | G | A | 0.16 | Nonsense |
| P045_P3 | chr15 | 43493041 | TP53BP1 | C | T | 0.15 | CdsStartSNV |
| P045_P3 | chr17 | 39501261 | CDK12 | C | T | 0.36 | Missense |
| P045_P3 | chr19 | 50409150 | POLD1 | C | A | 0.3 | Missense |
| P045_P4 | chr2 | 47790958 | MSH6 | G | A | 0.62 | Missense |
| P045_P4 | chr3 | 121539469 | POLQ | T | C | 2.41 | Missense |
| P045_P4 | chr13 | 48465206 | RB1 | C | T | 0.29 | Missense |
| P045_P4 | chr15 | 28117138 | HERC2 | C | T | 0.11 | Missense |
| P045_P4 | chr15 | 43447433 | TP53BP1 | C | A | 0.88 | Missense |
| P045_P5 | chr15 | 28177015 | HERC2 | A | T | 0.08 | Missense |
| P045_P5 | chr15 | 28198745 | HERC2 | G | A | 0.1 | Nonsense |
| P045_P5 | chr15 | 43493041 | TP53BP1 | C | T | 0.07 | CdsStartSNV |
| P045_P5 | chr16 | 56758587 | NUP93 | C | T | 0.05 | Nonsense |
| P045_P5 | chr17 | 39501261 | CDK12 | C | T | 0.12 | Missense |
| P045_P5 | chr17 | 39511533 | CDK12 | C | T | 0.19 | Missense |
| P045_P5 | chr19 | 50409150 | POLD1 | C | A | 0.05 | Missense |
| P045_tumour | chr2 | 47799146 | MSH6 | A | T | 3.66 | Missense |
| P045_tumour | chr3 | 121493478 | POLQ | C | T | 2.5 | Missense |
| P045_tumour | chr11 | 94429930 | MRE11 | A | C | 2.52 | Missense |
| P045_tumour | chr12 | 132661157 | POLE | C | A | 0.98 | Missense |
| P045_tumour | chr13 | 48465206 | RB1 | C | T | 1.06 | Missense |
| P045_tumour | chr17 | 39494623 | CDK12 | A | C | 0.53 | Missense |
| P045_tumour | chr17 | 39501259 | CDK12 | A | C | 0.61 | Missense |
| P045_tumour | chr17 | 39501261 | CDK12 | C | T | 1.11 | Missense |
| P045_tumour | chr17 | 39511533 | CDK12 | C | T | 0.36 | Missense |
| P045_tumour | chr17 | 39511546 | CDK12 | A | C | 0.36 | Missense |
| P045_tumour | chr22 | 28734705 | CHEK2 | T | A | 2.63 | Missense |
| P050_P1 | chr1 | 45332248 | MUTYH | T | C | 0.36 | Missense |
| P050_P1 | chr2 | 47800364 | MSH6 | C | T | 0.5 | Missense |
| P050_P1 | chr2 | 47800965 | MSH6 | C | A | 0.64 | Nonsense |
| P050_P1 | chr2 | 47801017 | MSH6 | G | T | 0.67 | Nonsense |
| P050_P1 | chr2 | 47804955 | MSH6 | G | A | 1.26 | Missense |
| P050_P1 | chr2 | 127280508 | ERCC3 | T | C | 0.23 | Missense |
| P050_P1 | chr2 | 127286985 | ERCC3 | C | T | 0.24 | Missense |
| P050_P1 | chr3 | 10074587 | FANCD2 | T | C | 0.36 | Missense |
| P050_P1 | chr3 | 10074664 | FANCD2 | G | A | 0.39 | Missense |
| P050_P1 | chr3 | 14147961 | XPC | G | A | 0.8 | Missense |
| P050_P1 | chr3 | 14148701 | XPC | G | T | 0.35 | Missense |
| P050_P1 | chr3 | 14148863 | XPC | C | T | 0.23 | Missense |
| P050_P1 | chr3 | 14148890 | XPC | C | A | 0.16 | Missense |
| P050_P1 | chr3 | 14158234 | XPC | C | T | 0.29 | Missense |
| P050_P1 | chr3 | 36993600 | MLH1 | G | A | 1.05 | Missense |
| P050_P1 | chr3 | 37007057 | MLH1 | G | T | 0.26 | Missense |
| P050_P1 | chr3 | 37020404 | MLH1 | C | T | 0.38 | Nonsense |
| P050_P1 | chr3 | 52408058 | BAP1 | G | A | 0.3 | Missense |
| P050_P1 | chr3 | 121467536 | POLQ | G | A | 0.43 | Missense |
| P050_P1 | chr3 | 121496832 | POLQ | G | A | 0.52 | Nonsense |
| P050_P1 | chr3 | 121539483 | POLQ | G | A | 0.66 | Missense |
| P050_P1 | chr3 | 121545865 | POLQ | G | A | 0.34 | Missense |
| P050_P1 | chr3 | 136421113 | STAG1 | C | A | 0.25 | Missense |
| P050_P1 | chr3 | 136500245 | STAG1 | C | A | 0.41 | Missense |
| P050_P1 | chr3 | 142457725 | ATR | G | A | 0.33 | Missense |
| P050_P1 | chr3 | 142459285 | ATR | T | C | 0.71 | Missense |
| P050_P1 | chr3 | 142465138 | ATR | T | C | 0.28 | Missense |
| P050_P1 | chr3 | 142542731 | ATR | C | A | 0.67 | Missense |
| P050_P1 | chr3 | 142550230 | ATR | G | A | 0.36 | Nonsense |
| P050_P1 | chr4 | 1904331 | NSD2 | G | A | 0.6 | Missense |
| P050_P1 | chr4 | 1974867 | NSD2 | G | A | 0.38 | Missense |
| P050_P1 | chr4 | 1974882 | NSD2 | G | A | 0.49 | Missense |
| P050_P1 | chr4 | 1978658 | NSD2 | C | A | 0.35 | Missense |
| P050_P1 | chr4 | 57010389 | POLR2B | C | A | 0.25 | Missense |
| P050_P1 | chr4 | 57023417 | POLR2B | G | A | 0.2 | Missense |
| P050_P1 | chr5 | 132640757 | RAD50 | C | A | 0.37 | Missense |
| P050_P1 | chr5 | 143207291 | ARHGAP26 | C | A | 0.11 | Missense |
| P050_P1 | chr6 | 35462832 | FANCE | T | C | 0.17 | Missense |
| P050_P1 | chr6 | 35466340 | FANCE | C | A | 0.23 | CdsStopSNV |
| P050_P1 | chr7 | 5977722 | PMS2 | G | A | 0.24 | Missense |
| P050_P1 | chr7 | 72926861 | POM121 | G | T | 0.28 | Missense |
| P050_P1 | chr9 | 35075718 | FANCG | G | A | 0.42 | Missense |
| P050_P1 | chr9 | 35078209 | FANCG | C | T | 0.32 | Missense |
| P050_P1 | chr9 | 95126575 | FANCC | C | T | 0.68 | Missense |
| P050_P1 | chr9 | 107323992 | RAD23B | G | A | 0.15 | Missense |
| P050_P1 | chr9 | 130862832 | ABL1 | G | A | 0.13 | Missense |
| P050_P1 | chr9 | 130862898 | ABL1 | T | C | 0.28 | Missense |
| P050_P1 | chr9 | 130862965 | ABL1 | G | A | 0.4 | Missense |
| P050_P1 | chr9 | 130874966 | ABL1 | C | T | 0.43 | Missense |
| P050_P1 | chr9 | 130878474 | ABL1 | G | A | 0.21 | Missense |
| P050_P1 | chr9 | 130884437 | ABL1 | C | A | 0.58 | Missense |
| P050_P1 | chr10 | 49472457 | ERCC6 | G | A | 1.02 | Missense |
| P050_P1 | chr10 | 49524325 | ERCC6 | C | A | 0.2 | Missense |
| P050_P1 | chr10 | 49532618 | ERCC6 | G | A | 0.13 | Missense |
| P050_P1 | chr10 | 68765990 | CCAR1 | G | A | 0.32 | Missense |
| P050_P1 | chr10 | 87513204 | MINPP1 | G | T | 0.29 | Missense |
| P050_P1 | chr10 | 129759244 | MGMT | T | C | 0.16 | Missense |
| P050_P1 | chr11 | 3676559 | NUP98 | C | A | 0.43 | Missense |
| P050_P1 | chr11 | 3712564 | NUP98 | C | A | 0.78 | Missense |
| P050_P1 | chr11 | 22624961 | FANCF | G | A | 0.45 | Missense |
| P050_P1 | chr11 | 22625279 | FANCF | C | A | 0.16 | Missense |
| P050_P1 | chr11 | 22625386 | FANCF | G | A | 0.36 | Missense |
| P050_P1 | chr11 | 47215231 | DDB2 | C | A | 0.49 | Missense |
| P050_P1 | chr11 | 61796001 | FEN1 | C | T | 0.31 | Nonsense |
| P050_P1 | chr11 | 108227801 | ATM | T | C | 0.95 | Missense |
| P050_P1 | chr11 | 108325352 | ATM | G | A | 0.28 | Nonsense |
| P050_P1 | chr11 | 108325416 | ATM | C | T | 0.36 | Missense |
| P050_P1 | chr11 | 108365413 | ATM | C | T | 1.03 | Missense |
| P050_P1 | chr12 | 68721152 | NUP107 | C | T | 0.69 | Missense |
| P050_P1 | chr12 | 68726565 | NUP107 | G | T | 0.26 | Missense |
| P050_P1 | chr12 | 132626268 | POLE | C | A | 0.17 | Missense |
| P050_P1 | chr12 | 132639219 | POLE | G | A | 0.38 | Missense |
| P050_P1 | chr12 | 132642295 | POLE | C | T | 0.94 | Nonsense |
| P050_P1 | chr12 | 132661093 | POLE | A | G | 1.03 | Missense |
| P050_P1 | chr12 | 132672787 | POLE | G | A | 0.71 | Missense |
| P050_P1 | chr12 | 132673661 | POLE | T | C | 0.29 | Missense |
| P050_P1 | chr12 | 132676631 | POLE | T | A | 0.76 | Missense |
| P050_P1 | chr13 | 32355210 | BRCA2 | G | T | 0.27 | Nonsense |
| P050_P1 | chr13 | 32363471 | BRCA2 | G | T | 0.47 | Nonsense |
| P050_P1 | chr13 | 48342675 | RB1 | C | A | 0.42 | Nonsense |
| P050_P1 | chr13 | 102862084 | ERCC5 | G | A | 0.42 | Missense |
| P050_P1 | chr15 | 28111905 | HERC2 | C | T | 0.47 | Missense |
| P050_P1 | chr15 | 28111914 | HERC2 | A | G | 0.26 | Missense |
| P050_P1 | chr15 | 28111918 | HERC2 | C | T | 0.19 | Missense |
| P050_P1 | chr15 | 28117036 | HERC2 | C | T | 0.18 | Missense |
| P050_P1 | chr15 | 28117051 | HERC2 | A | G | 0.25 | Missense |
| P050_P1 | chr15 | 28124123 | HERC2 | C | A | 0.48 | Missense |
| P050_P1 | chr15 | 28130552 | HERC2 | C | A | 0.51 | Nonsense |
| P050_P1 | chr15 | 28132158 | HERC2 | T | C | 0.45 | Missense |
| P050_P1 | chr15 | 28143988 | HERC2 | G | A | 3.93 | Missense |
| P050_P1 | chr15 | 28144100 | HERC2 | G | A | 0.16 | Missense |
| P050_P1 | chr15 | 28167713 | HERC2 | C | T | 0.13 | Missense |
| P050_P1 | chr15 | 28177059 | HERC2 | C | T | 1.63 | Missense |
| P050_P1 | chr15 | 28177096 | HERC2 | G | T | 0.26 | Missense |
| P050_P1 | chr15 | 28178971 | HERC2 | C | T | 0.19 | Missense |
| P050_P1 | chr15 | 28186697 | HERC2 | C | A | 0.35 | Missense |
| P050_P1 | chr15 | 28198711 | HERC2 | C | T | 0.53 | Missense |
| P050_P1 | chr15 | 28213882 | HERC2 | G | A | 0.26 | Missense |
| P050_P1 | chr15 | 28213914 | HERC2 | G | A | 0.15 | Missense |
| P050_P1 | chr15 | 28228256 | HERC2 | C | T | 0.39 | Missense |
| P050_P1 | chr15 | 28246813 | HERC2 | G | A | 0.31 | Missense |
| P050_P1 | chr15 | 28256125 | HERC2 | G | A | 0.39 | Missense |
| P050_P1 | chr15 | 28263106 | HERC2 | C | T | 0.16 | Missense |
| P050_P1 | chr15 | 28265861 | HERC2 | T | C | 0.92 | Missense |
| P050_P1 | chr15 | 28270736 | HERC2 | G | A | 0.61 | Missense |
| P050_P1 | chr15 | 28274301 | HERC2 | CG | TG | 0.37 | Missense |
| P050_P1 | chr15 | 28274911 | HERC2 | G | A | 0.2 | Nonsense |
| P050_P1 | chr15 | 28274962 | HERC2 | C | A | 0.17 | Nonsense |
| P050_P1 | chr15 | 34874708 | AQR | C | A | 0.19 | Missense |
| P050_P1 | chr15 | 34875979 | AQR | C | T | 0.74 | Missense |
| P050_P1 | chr15 | 34910231 | AQR | C | T | 0.33 | Missense |
| P050_P1 | chr15 | 34915135 | AQR | G | A | 0.36 | Missense |
| P050_P1 | chr15 | 43415746 | TP53BP1 | G | A | 0.24 | Missense |
| P050_P1 | chr15 | 43415783 | TP53BP1 | G | A | 0.27 | Missense |
| P050_P1 | chr15 | 43422015 | TP53BP1 | C | T | 0.28 | Missense |
| P050_P1 | chr15 | 43432346 | TP53BP1 | C | T | 0.26 | Missense |
| P050_P1 | chr15 | 43456723 | TP53BP1 | C | A | 0.31 | Missense |
| P050_P1 | chr15 | 43475617 | TP53BP1 | G | A | 0.55 | Missense |
| P050_P1 | chr15 | 43493043 | TP53BP1 | T | C | 0.17 | CdsStartSNV |
| P050_P1 | chr15 | 49133968 | COPS2 | G | A | 1 | Missense |
| P050_P1 | chr15 | 89261865 | FANCI | C | A | 1.28 | Missense |
| P050_P1 | chr15 | 89283147 | FANCI | C | T | 0.48 | Missense |
| P050_P1 | chr15 | 90782869 | BLM | C | A | 0.65 | Missense |
| P050_P1 | chr16 | 13935704 | ERCC4 | T | C | 0.52 | Missense |
| P050_P1 | chr16 | 13947902 | ERCC4 | T | C | 0.15 | Missense |
| P050_P1 | chr16 | 13947982 | ERCC4 | C | T | 0.1 | Missense |
| P050_P1 | chr16 | 56837627 | NUP93 | A | G | 0.21 | Missense |
| P050_P1 | chr16 | 89740065 | FANCA | C | A | 0.19 | Missense |
| P050_P1 | chr16 | 89746687 | FANCA | C | T | 0.36 | Missense |
| P050_P1 | chr16 | 89764963 | FANCA | T | A | 0.14 | Missense |
| P050_P1 | chr17 | 39490630 | CDK12 | C | A | 0.27 | Missense |
| P050_P1 | chr17 | 43045778 | BRCA1 | G | A | 0.5 | Missense |
| P050_P1 | chr17 | 43094627 | BRCA1 | C | T | 0.44 | Missense |
| P050_P1 | chr17 | 61743114 | BRIP1 | C | T | 0.3 | Missense |
| P050_P1 | chr17 | 61743116 | BRIP1 | G | A | 0.3 | Missense |
| P050_P1 | chr19 | 14106785 | PRKACA | G | A | 0.44 | Missense |
| P050_P1 | chr19 | 45352645 | ERCC2 | C | A | 0.16 | Missense |
| P050_P1 | chr19 | 45355715 | ERCC2 | C | T | 0.54 | Missense |
| P050_P1 | chr19 | 45357524 | ERCC2 | C | T | 0.14 | Missense |
| P050_P1 | chr19 | 45361538 | ERCC2 | C | T | 0.48 | Missense |
| P050_P1 | chr19 | 45364322 | ERCC2 | C | T | 0.32 | Missense |
| P050_P1 | chr19 | 45364869 | ERCC2 | C | T | 0.35 | Missense |
| P050_P1 | chr19 | 45364923 | ERCC2 | A | G | 0.2 | Missense |
| P050_P1 | chr19 | 45413965 | ERCC1 | C | A | 0.36 | Nonsense |
| P050_P1 | chr19 | 45414907 | ERCC1 | G | A | 0.19 | Missense |
| P050_P1 | chr19 | 45414926 | ERCC1 | T | G | 0.21 | Missense |
| P050_P1 | chr19 | 45420426 | ERCC1 | C | A | 0.33 | Missense |
| P050_P1 | chr19 | 50401875 | POLD1 | C | A | 0.11 | Nonsense |
| P050_P1 | chr19 | 50401891 | POLD1 | T | C | 0.12 | Missense |
| P050_P1 | chr19 | 50407346 | POLD1 | T | C | 0.1 | Missense |
| P050_P1 | chr19 | 50408792 | POLD1 | G | T | 0.36 | Missense |
| P050_P1 | chr19 | 50409178 | POLD1 | C | A | 0.19 | Nonsense |
| P050_P1 | chr19 | 50413436 | POLD1 | G | A | 0.21 | Missense |
| P050_P1 | chr19 | 50413767 | POLD1 | T | C | 0.16 | Missense |
| P050_P1 | chr19 | 50413771 | POLD1 | G | A | 0.16 | Missense |
| P050_P1 | chr20 | 63690161 | RTEL1 | G | A | 0.12 | Missense |
| P050_P1 | chr20 | 63690421 | RTEL1 | G | T | 0.32 | Missense |
| P050_P1 | chr22 | 41168823 | EP300 | G | A | 0.39 | Missense |
| P050_P1 | chr22 | 41176353 | EP300 | C | T | 0.6 | Missense |
| P050_P1 | chr22 | 41176969 | EP300 | G | A | 0.44 | Missense |
| P050_P1 | chr22 | 41177023 | EP300 | G | A | 0.15 | Missense |
| P050_P1 | chr22 | 41177502 | EP300 | C | T | 0.16 | Nonsense |
| P050_P1 | chr22 | 41178640 | EP300 | C | A | 0.13 | Missense |
| P050_P1 | chr22 | 41178660 | EP300 | C | T | 0.24 | Missense |
| P050_P1 | chrX | 53382353 | SMC1A | G | A | 0.38 | Missense |
| P050_P1 | chrX | 53382370 | SMC1A | G | T | 0.3 | Missense |
| P050_P1 | chrX | 53403617 | SMC1A | C | T | 0.36 | Missense |
| P050_P1 | chr1 | 45332605 | MUTYH | G | A | 0.06 | Missense |
| P050_P1 | chr1 | 45332827 | MUTYH | T | G | 0.03 | Missense |
| P050_P1 | chr2 | 127288674 | ERCC3 | A | G | 0.06 | Missense |
| P050_P1 | chr3 | 10067315 | FANCD2 | C | A | 0.14 | Missense |
| P050_P1 | chr3 | 10085877 | FANCD2 | G | T | 0.12 | Missense |
| P050_P1 | chr3 | 52405153 | BAP1 | G | T | 0.05 | Missense |
| P050_P1 | chr3 | 121545847 | POLQ | G | A | 0.08 | Missense |
| P050_P1 | chr3 | 136433605 | STAG1 | C | A | 0.2 | Missense |
| P050_P1 | chr3 | 142556485 | ATR | G | A | 0.06 | Missense |
| P050_P1 | chr4 | 1976506 | NSD2 | G | A | 0.09 | Missense |
| P050_P1 | chr4 | 1978644 | NSD2 | G | T | 0.18 | Missense |
| P050_P1 | chr4 | 1978787 | NSD2 | C | A | 0.22 | Missense |
| P050_P1 | chr9 | 35076777 | FANCG | G | A | 0.04 | Nonsense |
| P050_P1 | chr9 | 130862934 | ABL1 | G | A | 0.05 | Missense |
| P050_P1 | chr9 | 130885423 | ABL1 | A | T | 0.12 | Missense |
| P050_P1 | chr9 | 130885592 | ABL1 | C | T | 0.09 | Missense |
| P050_P1 | chr11 | 3683326 | NUP98 | A | G | 0.06 | Missense |
| P050_P1 | chr12 | 132643258 | POLE | G | T | 0.1 | Nonsense |
| P050_P1 | chr12 | 132643516 | POLE | A | T | 0.05 | Nonsense |
| P050_P1 | chr12 | 132657356 | POLE | A | G | 0.07 | Missense |
| P050_P1 | chr12 | 132673210 | POLE | G | A | 0.23 | Missense |
| P050_P1 | chr15 | 28117148 | HERC2 | G | A | 0.05 | Nonsense |
| P050_P1 | chr15 | 28141493 | HERC2 | A | G | 0.19 | Missense |
| P050_P1 | chr15 | 28144801 | HERC2 | C | A | 0.07 | Missense |
| P050_P1 | chr15 | 28177036 | HERC2 | A | G | 0.05 | Missense |
| P050_P1 | chr15 | 28198711 | HERC2 | C | A | 0.53 | Missense |
| P050_P1 | chr15 | 28213809 | HERC2 | C | T | 0.06 | Missense |
| P050_P1 | chr15 | 43415632 | TP53BP1 | G | A | 0.06 | Missense |
| P050_P1 | chr15 | 49128084 | COPS2 | C | A | 0.08 | Missense |
| P050_P1 | chr16 | 56834248 | NUP93 | T | C | 0.06 | Missense |
| P050_P1 | chr17 | 35106459 | RAD51D | T | G | 0.78 | Missense |
| P050_P1 | chr17 | 35107053 | RAD51D | C | A | 0.07 | Nonsense |
| P050_P1 | chr17 | 39531044 | CDK12 | C | T | 0.06 | Nonsense |
| P050_P1 | chr19 | 50402213 | POLD1 | G | T | 0.07 | Missense |
| P050_P1 | chr20 | 63690200 | RTEL1 | C | T | 0.04 | Missense |
| P050_P1 | chr22 | 41178241 | EP300 | C | A | 0.04 | Nonsense |
| P050_P2 | chr2 | 127280508 | ERCC3 | T | C | 0.22 | Missense |
| P050_P2 | chr2 | 127286985 | ERCC3 | C | T | 0.07 | Missense |
| P050_P2 | chr3 | 10074664 | FANCD2 | G | A | 0.46 | Missense |
| P050_P2 | chr3 | 14148890 | XPC | C | A | 0.23 | Missense |
| P050_P2 | chr3 | 14158234 | XPC | C | T | 0.41 | Missense |
| P050_P2 | chr3 | 36993600 | MLH1 | G | A | 0.17 | Missense |
| P050_P2 | chr3 | 52408058 | BAP1 | G | A | 0.09 | Missense |
| P050_P2 | chr3 | 121496832 | POLQ | G | A | 0.08 | Nonsense |
| P050_P2 | chr3 | 121545865 | POLQ | G | A | 0.11 | Missense |
| P050_P2 | chr3 | 142550230 | ATR | G | A | 0.12 | Nonsense |
| P050_P2 | chr4 | 1904331 | NSD2 | G | A | 0.3 | Missense |
| P050_P2 | chr4 | 1974867 | NSD2 | G | A | 0.55 | Missense |
| P050_P2 | chr4 | 1974882 | NSD2 | G | A | 0.14 | Missense |
| P050_P2 | chr4 | 1978658 | NSD2 | C | A | 0.07 | Missense |
| P050_P2 | chr4 | 57023417 | POLR2B | G | A | 0.22 | Missense |
| P050_P2 | chr5 | 143207291 | ARHGAP26 | C | A | 0.1 | Missense |
| P050_P2 | chr7 | 72926861 | POM121 | G | T | 0.05 | Missense |
| P050_P2 | chr9 | 95126575 | FANCC | C | T | 0.12 | Missense |
| P050_P2 | chr9 | 130862832 | ABL1 | G | A | 0.54 | Missense |
| P050_P2 | chr9 | 130862965 | ABL1 | G | A | 0.25 | Missense |
| P050_P2 | chr9 | 130884437 | ABL1 | C | A | 0.15 | Missense |
| P050_P2 | chr10 | 49524325 | ERCC6 | C | A | 0.04 | Missense |
| P050_P2 | chr10 | 49532618 | ERCC6 | G | A | 0.22 | Missense |
| P050_P2 | chr11 | 3676559 | NUP98 | C | A | 0.48 | Missense |
| P050_P2 | chr11 | 61796001 | FEN1 | C | T | 0.48 | Nonsense |
| P050_P2 | chr11 | 108325352 | ATM | G | A | 0.39 | Nonsense |
| P050_P2 | chr11 | 108325416 | ATM | C | T | 0.3 | Missense |
| P050_P2 | chr11 | 108365413 | ATM | C | T | 0.4 | Missense |
| P050_P2 | chr12 | 68721152 | NUP107 | C | T | 0.21 | Missense |
| P050_P2 | chr12 | 132626268 | POLE | C | A | 0.17 | Missense |
| P050_P2 | chr12 | 132639219 | POLE | G | A | 0.06 | Missense |
| P050_P2 | chr12 | 132642295 | POLE | C | T | 0.27 | Nonsense |
| P050_P2 | chr12 | 132661093 | POLE | A | G | 0.64 | Missense |
| P050_P2 | chr12 | 132672787 | POLE | G | A | 0.07 | Missense |
| P050_P2 | chr13 | 32355210 | BRCA2 | G | T | 0.22 | Nonsense |
| P050_P2 | chr13 | 48342675 | RB1 | C | A | 0.08 | Nonsense |
| P050_P2 | chr13 | 102862084 | ERCC5 | G | A | 0.21 | Missense |
| P050_P2 | chr15 | 28111905 | HERC2 | C | T | 0.58 | Missense |
| P050_P2 | chr15 | 28111914 | HERC2 | A | G | 0.11 | Missense |
| P050_P2 | chr15 | 28144100 | HERC2 | G | A | 0.18 | Missense |
| P050_P2 | chr15 | 28167713 | HERC2 | C | T | 0.74 | Missense |
| P050_P2 | chr15 | 28177059 | HERC2 | C | T | 0.05 | Missense |
| P050_P2 | chr15 | 28178971 | HERC2 | C | T | 0.11 | Missense |
| P050_P2 | chr15 | 28198711 | HERC2 | C | T | 0.17 | Missense |
| P050_P2 | chr15 | 28213882 | HERC2 | G | A | 0.07 | Missense |
| P050_P2 | chr15 | 28213914 | HERC2 | G | A | 0.06 | Missense |
| P050_P2 | chr15 | 28256125 | HERC2 | G | A | 0.13 | Missense |
| P050_P2 | chr15 | 28263106 | HERC2 | C | T | 0.21 | Missense |
| P050_P2 | chr15 | 28265861 | HERC2 | T | C | 0.72 | Missense |
| P050_P2 | chr15 | 28274301 | HERC2 | CG | TG | 0.11 | Missense |
| P050_P2 | chr15 | 28274911 | HERC2 | G | A | 0.33 | Nonsense |
| P050_P2 | chr15 | 43475617 | TP53BP1 | G | A | 1.09 | Missense |
| P050_P2 | chr15 | 49133968 | COPS2 | G | A | 0.14 | Missense |
| P050_P2 | chr15 | 90782869 | BLM | C | A | 0.57 | Missense |
| P050_P2 | chr16 | 13935704 | ERCC4 | T | C | 0.18 | Missense |
| P050_P2 | chr16 | 13947902 | ERCC4 | T | C | 0.05 | Missense |
| P050_P2 | chr16 | 13947982 | ERCC4 | C | T | 0.04 | Missense |
| P050_P2 | chr16 | 56837627 | NUP93 | A | G | 0.12 | Missense |
| P050_P2 | chr17 | 43045778 | BRCA1 | G | A | 0.08 | Missense |
| P050_P2 | chr17 | 61743114 | BRIP1 | C | T | 0.08 | Missense |
| P050_P2 | chr19 | 45357524 | ERCC2 | C | T | 0.06 | Missense |
| P050_P2 | chr19 | 45364923 | ERCC2 | A | G | 0.07 | Missense |
| P050_P2 | chr19 | 45414907 | ERCC1 | G | A | 0.11 | Missense |
| P050_P2 | chr19 | 50401875 | POLD1 | C | A | 0.37 | Nonsense |
| P050_P2 | chr19 | 50408792 | POLD1 | G | T | 0.11 | Missense |
| P050_P2 | chr19 | 50409178 | POLD1 | C | A | 0.09 | Nonsense |
| P050_P2 | chr19 | 50413771 | POLD1 | G | A | 0.17 | Missense |
| P050_P2 | chr20 | 63690161 | RTEL1 | G | A | 0.26 | Missense |
| P050_P2 | chr22 | 41176353 | EP300 | C | T | 0.14 | Missense |
| P050_P2 | chr22 | 41177502 | EP300 | C | T | 0.11 | Nonsense |
| P050_P2 | chrX | 53382370 | SMC1A | G | T | 0.1 | Missense |
| P050_P2 | chr1 | 35743229 | CLSPN | C | A | 0.17 | Missense |
| P050_P2 | chr1 | 45331671 | MUTYH | C | G | 0.13 | Missense |
| P050_P2 | chr1 | 45332605 | MUTYH | G | A | 0.13 | Missense |
| P050_P2 | chr1 | 45332827 | MUTYH | T | G | 0.12 | Missense |
| P050_P2 | chr2 | 47798945 | MSH6 | C | A | 0.63 | Nonsense |
| P050_P2 | chr2 | 47799493 | MSH6 | A | T | 0.31 | Nonsense |
| P050_P2 | chr2 | 47800445 | MSH6 | T | C | 0.17 | Missense |
| P050_P2 | chr2 | 47806456 | MSH6 | G | A | 0.51 | Missense |
| P050_P2 | chr2 | 127288674 | ERCC3 | A | G | 0.14 | Missense |
| P050_P2 | chr2 | 214745732 | BARD1 | A | C | 0.3 | Missense |
| P050_P2 | chr2 | 214781099 | BARD1 | C | A | 0.45 | Missense |
| P050_P2 | chr3 | 10063877 | FANCD2 | T | C | 0.39 | Missense |
| P050_P2 | chr3 | 10065447 | FANCD2 | T | G | 0.34 | Missense |
| P050_P2 | chr3 | 10067315 | FANCD2 | C | A | 0.22 | Missense |
| P050_P2 | chr3 | 10085877 | FANCD2 | G | T | 0.21 | Missense |
| P050_P2 | chr3 | 37007025 | MLH1 | C | A | 0.28 | Missense |
| P050_P2 | chr3 | 37012051 | MLH1 | C | T | 0.29 | Missense |
| P050_P2 | chr3 | 37012099 | MLH1 | G | A | 0.34 | Missense |
| P050_P2 | chr3 | 37020324 | MLH1 | C | T | 0.12 | Missense |
| P050_P2 | chr3 | 37020339 | MLH1 | T | A | 0.11 | Missense |
| P050_P2 | chr3 | 52405153 | BAP1 | G | T | 0.11 | Missense |
| P050_P2 | chr3 | 121473361 | POLQ | T | A | 0.22 | Missense |
| P050_P2 | chr3 | 121545847 | POLQ | G | A | 0.46 | Missense |
| P050_P2 | chr3 | 136433605 | STAG1 | C | A | 0.25 | Missense |
| P050_P2 | chr3 | 142556485 | ATR | G | A | 0.37 | Missense |
| P050_P2 | chr3 | 142556488 | ATR | C | T | 0.25 | Nonsense |
| P050_P2 | chr3 | 142568087 | ATR | G | A | 0.73 | Missense |
| P050_P2 | chr3 | 186792834 | RFC4 | C | A | 0.5 | Missense |
| P050_P2 | chr3 | 186801121 | RFC4 | G | A | 0.84 | Missense |
| P050_P2 | chr4 | 1956090 | NSD2 | A | G | 0.35 | Missense |
| P050_P2 | chr4 | 1959616 | NSD2 | A | G | 0.17 | Missense |
| P050_P2 | chr4 | 1976506 | NSD2 | G | A | 0.48 | Missense |
| P050_P2 | chr4 | 1976578 | NSD2 | G | A | 0.34 | Missense |
| P050_P2 | chr4 | 1978643 | NSD2 | T | C | 0.28 | Missense |
| P050_P2 | chr4 | 1978644 | NSD2 | G | T | 0.19 | Missense |
| P050_P2 | chr4 | 1978787 | NSD2 | C | A | 0.11 | Missense |
| P050_P2 | chr4 | 57005622 | POLR2B | C | A | 0.33 | Missense |
| P050_P2 | chr4 | 57024011 | POLR2B | C | T | 0.42 | Missense |
| P050_P2 | chr4 | 57030257 | POLR2B | G | T | 0.22 | Missense |
| P050_P2 | chr5 | 132557416 | RAD50 | T | C | 0.63 | Missense |
| P050_P2 | chr5 | 132638204 | RAD50 | G | T | 0.53 | Missense |
| P050_P2 | chr6 | 35460614 | FANCE | G | T | 0.23 | Missense |
| P050_P2 | chr6 | 35466302 | FANCE | T | C | 0.13 | Missense |
| P050_P2 | chr7 | 45592765 | ADCY1 | G | A | 0.27 | Missense |
| P050_P2 | chr7 | 124835334 | POT1 | C | A | 0.28 | Nonsense |
| P050_P2 | chr8 | 116848966 | RAD21 | G | A | 0.26 | Nonsense |
| P050_P2 | chr9 | 35076777 | FANCG | G | A | 0.26 | Nonsense |
| P050_P2 | chr9 | 35077318 | FANCG | G | A | 0.21 | Nonsense |
| P050_P2 | chr9 | 95111513 | FANCC | C | T | 0.21 | Missense |
| P050_P2 | chr9 | 95125166 | FANCC | C | A | 0.36 | Missense |
| P050_P2 | chr9 | 97675569 | XPA | C | A | 0.27 | Missense |
| P050_P2 | chr9 | 130862934 | ABL1 | G | A | 0.13 | Missense |
| P050_P2 | chr9 | 130885423 | ABL1 | A | T | 0.13 | Missense |
| P050_P2 | chr9 | 130885547 | ABL1 | C | T | 0.29 | Missense |
| P050_P2 | chr9 | 130885550 | ABL1 | T | C | 0.11 | Missense |
| P050_P2 | chr9 | 130885592 | ABL1 | C | T | 0.21 | Missense |
| P050_P2 | chr10 | 49470266 | ERCC6 | G | A | 0.14 | Nonsense |
| P050_P2 | chr10 | 49532637 | ERCC6 | G | A | 0.11 | Missense |
| P050_P2 | chr11 | 3683326 | NUP98 | A | G | 0.17 | Missense |
| P050_P2 | chr11 | 3683400 | NUP98 | C | T | 0.49 | Missense |
| P050_P2 | chr11 | 3720733 | NUP98 | C | A | 0.3 | Missense |
| P050_P2 | chr11 | 47235312 | DDB2 | A | G | 0.42 | Missense |
| P050_P2 | chr11 | 94460941 | MRE11 | C | A | 0.84 | Nonsense |
| P050_P2 | chr11 | 108293388 | ATM | G | T | 0.4 | Missense |
| P050_P2 | chr12 | 68710032 | NUP107 | G | A | 0.33 | Missense |
| P050_P2 | chr12 | 132624933 | POLE | C | A | 0.21 | Missense |
| P050_P2 | chr12 | 132641786 | POLE | C | A | 0.28 | Missense |
| P050_P2 | chr12 | 132643258 | POLE | G | T | 0.11 | Nonsense |
| P050_P2 | chr12 | 132643516 | POLE | A | T | 0.11 | Nonsense |
| P050_P2 | chr12 | 132657243 | POLE | G | A | 0.16 | Missense |
| P050_P2 | chr12 | 132657356 | POLE | A | G | 0.57 | Missense |
| P050_P2 | chr12 | 132659475 | POLE | A | T | 0.1 | Missense |
| P050_P2 | chr12 | 132672765 | POLE | G | T | 0.11 | Missense |
| P050_P2 | chr12 | 132672808 | POLE | T | C | 0.58 | Missense |
| P050_P2 | chr12 | 132673210 | POLE | G | A | 0.86 | Missense |
| P050_P2 | chr13 | 102853834 | ERCC5 | G | T | 0.46 | Missense |
| P050_P2 | chr13 | 102862059 | ERCC5 | G | T | 0.24 | Missense |
| P050_P2 | chr15 | 28113620 | HERC2 | G | A | 0.32 | Missense |
| P050_P2 | chr15 | 28113676 | HERC2 | A | G | 0.22 | Missense |
| P050_P2 | chr15 | 28117148 | HERC2 | G | A | 0.13 | Nonsense |
| P050_P2 | chr15 | 28125154 | HERC2 | A | T | 0.16 | Missense |
| P050_P2 | chr15 | 28130184 | HERC2 | A | G | 0.15 | Missense |
| P050_P2 | chr15 | 28130224 | HERC2 | C | A | 0.3 | Missense |
| P050_P2 | chr15 | 28141493 | HERC2 | A | G | 0.14 | Missense |
| P050_P2 | chr15 | 28141525 | HERC2 | T | A | 0.24 | Missense |
| P050_P2 | chr15 | 28144801 | HERC2 | C | A | 0.13 | Missense |
| P050_P2 | chr15 | 28174514 | HERC2 | C | A | 0.22 | Missense |
| P050_P2 | chr15 | 28176967 | HERC2 | G | A | 0.11 | Nonsense |
| P050_P2 | chr15 | 28177036 | HERC2 | A | G | 0.4 | Missense |
| P050_P2 | chr15 | 28178947 | HERC2 | T | A | 0.13 | Missense |
| P050_P2 | chr15 | 28178955 | HERC2 | G | A | 1.3 | Missense |
| P050_P2 | chr15 | 28198711 | HERC2 | C | A | 0.17 | Missense |
| P050_P2 | chr15 | 28213809 | HERC2 | C | T | 0.24 | Missense |
| P050_P2 | chr15 | 28263154 | HERC2 | C | A | 0.55 | Missense |
| P050_P2 | chr15 | 34890323 | AQR | G | A | 0.44 | Missense |
| P050_P2 | chr15 | 43408056 | TP53BP1 | T | A | 0.36 | Missense |
| P050_P2 | chr15 | 43415615 | TP53BP1 | T | C | 0.18 | Missense |
| P050_P2 | chr15 | 43415632 | TP53BP1 | G | A | 0.82 | Missense |
| P050_P2 | chr15 | 43420604 | TP53BP1 | G | T | 0.36 | Missense |
| P050_P2 | chr15 | 43421065 | TP53BP1 | C | A | 0.16 | Missense |
| P050_P2 | chr15 | 49128084 | COPS2 | C | A | 0.23 | Missense |
| P050_P2 | chr15 | 49134076 | COPS2 | C | A | 0.42 | Nonsense |
| P050_P2 | chr15 | 90763151 | BLM | C | T | 0.49 | Missense |
| P050_P2 | chr15 | 90790748 | BLM | C | A | 0.42 | Missense |
| P050_P2 | chr16 | 23626345 | PALB2 | G | A | 0.17 | Missense |
| P050_P2 | chr16 | 23629909 | PALB2 | C | A | 0.17 | Nonsense |
| P050_P2 | chr16 | 56829036 | NUP93 | G | T | 0.71 | Missense |
| P050_P2 | chr16 | 56834248 | NUP93 | T | C | 0.18 | Missense |
| P050_P2 | chr17 | 7673740 | TP53 | C | A | 0.11 | Nonsense |
| P050_P2 | chr17 | 7675071 | TP53 | G | A | 0.13 | Missense |
| P050_P2 | chr17 | 35101329 | RAD51D | T | A | 0.13 | Missense |
| P050_P2 | chr17 | 35106459 | RAD51D | T | G | 0.27 | Missense |
| P050_P2 | chr17 | 35107053 | RAD51D | C | A | 0.2 | Nonsense |
| P050_P2 | chr17 | 39490719 | CDK12 | T | A | 0.12 | Nonsense |
| P050_P2 | chr17 | 39501295 | CDK12 | G | A | 0.28 | Missense |
| P050_P2 | chr17 | 39511591 | CDK12 | A | G | 0.25 | Missense |
| P050_P2 | chr17 | 39531044 | CDK12 | C | T | 0.23 | Nonsense |
| P050_P2 | chr17 | 43092934 | BRCA1 | C | A | 0.21 | Missense |
| P050_P2 | chr17 | 60600541 | PPM1D | C | T | 0.51 | Missense |
| P050_P2 | chr17 | 61743091 | BRIP1 | C | A | 0.16 | Missense |
| P050_P2 | chr19 | 42287720 | CIC | C | T | 0.53 | Missense |
| P050_P2 | chr19 | 45352636 | ERCC2 | T | C | 0.1 | Missense |
| P050_P2 | chr19 | 45352796 | ERCC2 | C | T | 0.23 | Missense |
| P050_P2 | chr19 | 45370195 | ERCC2 | C | T | 0.31 | Missense |
| P050_P2 | chr19 | 45414001 | ERCC1 | T | C | 0.17 | Missense |
| P050_P2 | chr19 | 50402213 | POLD1 | G | T | 0.18 | Missense |
| P050_P2 | chr19 | 50413857 | POLD1 | C | T | 0.47 | Missense |
| P050_P2 | chr19 | 50414818 | POLD1 | T | C | 0.11 | Missense |
| P050_P2 | chr19 | 50417889 | POLD1 | T | C | 0.38 | Missense |
| P050_P2 | chr19 | 50417927 | POLD1 | C | A | 0.17 | Missense |
| P050_P2 | chr20 | 63680691 | RTEL1 | G | T | 0.1 | Missense |
| P050_P2 | chr20 | 63690200 | RTEL1 | C | T | 0.14 | Missense |
| P050_P2 | chr21 | 33755319 | ITSN1 | G | A | 0.23 | Missense |
| P050_P2 | chr22 | 41178241 | EP300 | C | A | 0.36 | Nonsense |
| P050_P2 | chr22 | 41178552 | EP300 | C | T | 0.12 | Nonsense |
| P050_P2 | chr22 | 41178679 | EP300 | C | T | 0.28 | Missense |
| P050_P2 | chrX | 124066401 | STAG2 | C | T | 0.38 | Nonsense |
| P095_P1 | chr10 | 49532618 | ERCC6 | G | A | 1.12 | Missense |
| P095_P1 | chr11 | 61796196 | FEN1 | G | A | 3.26 | Missense |
| P095_P1 | chr12 | 68719396 | NUP107 | G | A | 1.71 | Missense |
| P095_P1 | chr12 | 132668413 | POLE | C | T | 1.48 | Missense |
| P095_P1 | chr13 | 32338026 | BRCA2 | G | A | 1.3 | Missense |
| P095_P1 | chr15 | 28191159 | HERC2 | G | A | 1.61 | Missense |
| P095_P1 | chr16 | 56838957 | NUP93 | G | T | 0.76 | Missense |
| P095_P1 | chr16 | 89745063 | FANCA | C | T | 1.89 | Nonsense |
| P095_P1 | chr17 | 7676592 | TP53 | C | A | 1.63 | CdsStartSNV |
| P095_P1 | chr19 | 50416468 | POLD1 | C | T | 3.45 | Missense |
| P095_P2 | chr4 | 56999638 | POLR2B | G | T | 0.4 | Missense |
| P095_P2 | chr11 | 61796196 | FEN1 | G | A | 0.17 | Missense |
| P095_P2 | chr13 | 32338026 | BRCA2 | G | A | 0.52 | Missense |
| P095_P3 | chr10 | 49532618 | ERCC6 | G | A | 0.53 | Missense |
| P095_P3 | chr12 | 68719396 | NUP107 | G | A | 0.7 | Missense |
| P095_P4 | chr10 | 49532618 | ERCC6 | G | A | 0.16 | Missense |
| P095_P4 | chr13 | 32338026 | BRCA2 | G | A | 0.23 | Missense |
| P095_P4 | chr17 | 7676592 | TP53 | C | A | 0.21 | CdsStartSNV |
| P095_tumour | chr4 | 1959471 | NSD2 | G | T | 3.3 | Missense |
| P095_tumour | chr4 | 56999638 | POLR2B | G | T | 2.8 | Missense |
| P095_tumour | chr8 | 116856219 | RAD21 | G | T | 3.23 | Missense |
| P095_tumour | chr9 | 97693651 | XPA | G | T | 2.61 | Missense |
| P095_tumour | chr15 | 34882524 | AQR | T | A | 3 | Missense |
| P095_tumour | chr15 | 34893677 | AQR | T | A | 3.23 | Missense |
| P095_tumour | chr16 | 89745063 | FANCA | C | T | 0.71 | Nonsense |
| P095_tumour | chr17 | 7675076 | TP53 | T | C | 14.61 | Missense |
| P095_tumour | chr19 | 50416468 | POLD1 | C | T | 0.7 | Missense |
| P004_P1 | chr1 | 35739146 | CLSPN | C | A | 0.51 | Missense |
| P004_P1 | chr2 | 47480768 | MSH2 | C | T | 0.53 | Missense |
| P004_P1 | chr2 | 127259424 | ERCC3 | C | A | 0.23 | Nonsense |
| P004_P1 | chr2 | 127261307 | ERCC3 | A | G | 0.8 | Missense |
| P004_P1 | chr2 | 127288723 | ERCC3 | T | C | 0.26 | Missense |
| P004_P1 | chr3 | 14158238 | XPC | G | A | 0.35 | Missense |
| P004_P1 | chr3 | 52403839 | BAP1 | G | A | 0.59 | Nonsense |
| P004_P1 | chr3 | 136349298 | STAG1 | G | A | 0.86 | Missense |
| P004_P1 | chr4 | 1959631 | NSD2 | G | A | 0.48 | Missense |
| P004_P1 | chr4 | 1961090 | NSD2 | C | T | 0.42 | Missense |
| P004_P1 | chr4 | 1961104 | NSD2 | G | A | 0.35 | Missense |
| P004_P1 | chr4 | 57010416 | POLR2B | C | A | 0.25 | Missense |
| P004_P1 | chr4 | 57023492 | POLR2B | G | A | 0.59 | Missense |
| P004_P1 | chr7 | 5977755 | PMS2 | G | T | 0.24 | Missense |
| P004_P1 | chr9 | 130862766 | ABL1 | T | C | 0.34 | Missense |
| P004_P1 | chr9 | 130862830 | ABL1 | C | T | 0.26 | Missense |
| P004_P1 | chr9 | 130885301 | ABL1 | C | A | 0.24 | Missense |
| P004_P1 | chr10 | 49478354 | ERCC6 | C | A | 0.58 | Missense |
| P004_P1 | chr10 | 49532618 | ERCC6 | G | A | 0.38 | Missense |
| P004_P1 | chr10 | 129759262 | MGMT | T | C | 0.81 | Missense |
| P004_P1 | chr11 | 61795594 | FEN1 | G | A | 0.64 | Missense |
| P004_P1 | chr11 | 108272744 | ATM | C | A | 0.4 | Missense |
| P004_P1 | chr12 | 132667603 | POLE | T | C | 0.33 | Missense |
| P004_P1 | chr12 | 132673172 | POLE | G | A | 0.76 | Missense |
| P004_P1 | chr15 | 28132763 | HERC2 | C | A | 0.28 | Nonsense |
| P004_P1 | chr15 | 28142323 | HERC2 | G | A | 0.92 | Missense |
| P004_P1 | chr15 | 28152727 | HERC2 | C | A | 0.39 | Missense |
| P004_P1 | chr15 | 28174554 | HERC2 | G | A | 0.49 | Missense |
| P004_P1 | chr15 | 28176982 | HERC2 | C | A | 0.5 | Missense |
| P004_P1 | chr15 | 43421055 | TP53BP1 | C | T | 0.44 | Missense |
| P004_P1 | chr15 | 43421109 | TP53BP1 | C | T | 0.72 | Missense |
| P004_P1 | chr15 | 89261833 | FANCI | G | T | 1.01 | Missense |
| P004_P1 | chr16 | 23626345 | PALB2 | G | A | 0.39 | Missense |
| P004_P1 | chr16 | 23630185 | PALB2 | C | A | 1.04 | Nonsense |
| P004_P1 | chr16 | 56823786 | NUP93 | G | A | 0.34 | Missense |
| P004_P1 | chr16 | 56830531 | NUP93 | G | T | 0.46 | Nonsense |
| P004_P1 | chr17 | 7675208 | TP53 | C | T | 10.78 | Missense |
| P004_P1 | chr17 | 35103463 | RAD51D | G | A | 0.28 | Nonsense |
| P004_P1 | chr17 | 39471723 | CDK12 | C | T | 0.33 | Missense |
| P004_P1 | chr17 | 39494593 | CDK12 | G | A | 0.61 | Missense |
| P004_P1 | chr17 | 43082538 | BRCA1 | T | C | 0.8 | Missense |
| P004_P1 | chr17 | 61780329 | BRIP1 | A | G | 1.39 | Missense |
| P004_P1 | chr19 | 45352633 | ERCC2 | A | T | 0.22 | Missense |
| P004_P1 | chr19 | 45413637 | ERCC1 | C | A | 0.37 | Nonsense |
| P004_P1 | chr19 | 50406419 | POLD1 | G | T | 1.09 | Nonsense |
| P004_P1 | chr19 | 50406476 | POLD1 | C | T | 0.79 | Nonsense |
| P004_P1 | chr20 | 63688384 | RTEL1 | C | T | 0.56 | Missense |
| P004_P1 | chr22 | 41149966 | EP300 | C | A | 0.24 | Missense |
| P004_P1 | chr22 | 41173762 | EP300 | C | T | 0.8 | Missense |
| P004_P1 | chr22 | 41177530 | EP300 | G | A | 0.42 | Missense |
| P004_P1 | chr22 | 41178075 | EP300 | C | T | 0.52 | Nonsense |
| P004_P1 | chrX | 53405071 | SMC1A | T | C | 0.39 | Missense |
| P004_P1 | chrX | 124050206 | STAG2 | G | A | 1.75 | Missense |
| P051_P1 | chr1 | 35760462 | CLSPN | C | A | 1 | Nonsense |
| P051_P1 | chr1 | 45332469 | MUTYH | C | T | 0.45 | Missense |
| P051_P1 | chr1 | 45332953 | MUTYH | G | A | 0.47 | Missense |
| P051_P1 | chr1 | 147286302 | CHD1L | G | A | 0.72 | Missense |
| P051_P1 | chr2 | 47800855 | MSH6 | C | T | 0.82 | Nonsense |
| P051_P1 | chr3 | 14152378 | XPC | A | G | 0.25 | Missense |
| P051_P1 | chr3 | 14158156 | XPC | C | T | 0.39 | Missense |
| P051_P1 | chr3 | 52402779 | BAP1 | C | A | 0.61 | Missense |
| P051_P1 | chr3 | 136464905 | STAG1 | G | T | 0.41 | Missense |
| P051_P1 | chr3 | 142496477 | ATR | C | T | 0.42 | Missense |
| P051_P1 | chr3 | 142505192 | ATR | G | T | 0.47 | Missense |
| P051_P1 | chr3 | 142553883 | ATR | C | A | 0.45 | Missense |
| P051_P1 | chr3 | 142562819 | ATR | G | A | 0.8 | Nonsense |
| P051_P1 | chr4 | 1974867 | NSD2 | G | A | 0.3 | Missense |
| P051_P1 | chr4 | 1974984 | NSD2 | C | T | 0.36 | Missense |
| P051_P1 | chr4 | 57010430 | POLR2B | G | T | 0.41 | Missense |
| P051_P1 | chr6 | 35466295 | FANCE | A | C | 0.16 | Missense |
| P051_P1 | chr7 | 2924252 | CARD11 | T | A | 0.62 | Missense |
| P051_P1 | chr7 | 124863591 | POT1 | G | A | 0.78 | Missense |
| P051_P1 | chr8 | 31116470 | WRN | G | T | 0.87 | Missense |
| P051_P1 | chr8 | 31150372 | WRN | G | T | 0.36 | Missense |
| P051_P1 | chr8 | 31150388 | WRN | G | A | 0.2 | Missense |
| P051_P1 | chr8 | 31150394 | WRN | C | A | 0.47 | Missense |
| P051_P1 | chr8 | 116849004 | RAD21 | T | C | 0.68 | Missense |
| P051_P1 | chr9 | 21970928 | CDKN2A | C | T | 0.24 | Missense |
| P051_P1 | chr9 | 35075990 | FANCG | G | A | 0.45 | Missense |
| P051_P1 | chr9 | 35076984 | FANCG | C | T | 0.37 | Missense |
| P051_P1 | chr9 | 130862830 | ABL1 | C | T | 0.2 | Missense |
| P051_P1 | chr9 | 130862859 | ABL1 | C | A | 0.13 | Missense |
| P051_P1 | chr9 | 130862901 | ABL1 | C | A | 0.28 | Missense |
| P051_P1 | chr9 | 130874929 | ABL1 | G | T | 0.33 | Missense |
| P051_P1 | chr9 | 130874986 | ABL1 | C | A | 0.26 | Missense |
| P051_P1 | chr9 | 130878514 | ABL1 | G | A | 0.4 | Missense |
| P051_P1 | chr9 | 130884121 | ABL1 | G | A | 0.21 | Missense |
| P051_P1 | chr9 | 130884164 | ABL1 | A | G | 0.16 | Missense |
| P051_P1 | chr9 | 130885352 | ABL1 | G | T | 0.15 | Missense |
| P051_P1 | chr10 | 49470298 | ERCC6 | C | A | 0.45 | Missense |
| P051_P1 | chr11 | 3713976 | NUP98 | C | A | 0.47 | Missense |
| P051_P1 | chr11 | 22625084 | FANCF | C | T | 0.17 | Missense |
| P051_P1 | chr11 | 22625150 | FANCF | C | A | 0.45 | Nonsense |
| P051_P1 | chr11 | 108272602 | ATM | C | T | 1.64 | Missense |
| P051_P1 | chr11 | 108329190 | ATM | C | T | 0.99 | Missense |
| P051_P1 | chr12 | 68719342 | NUP107 | C | A | 0.73 | Missense |
| P051_P1 | chr12 | 132625662 | POLE | A | T | 0.48 | Missense |
| P051_P1 | chr12 | 132638044 | POLE | G | A | 0.21 | Missense |
| P051_P1 | chr12 | 132638051 | POLE | C | A | 0.2 | Nonsense |
| P051_P1 | chr12 | 132639236 | POLE | G | A | 0.54 | Missense |
| P051_P1 | chr12 | 132661630 | POLE | A | G | 0.53 | Missense |
| P051_P1 | chr12 | 132665411 | POLE | C | A | 1.09 | Missense |
| P051_P1 | chr12 | 132667558 | POLE | A | G | 0.25 | Missense |
| P051_P1 | chr12 | 132673249 | POLE | G | A | 0.59 | Missense |
| P051_P1 | chr15 | 28130195 | HERC2 | C | T | 0.49 | Missense |
| P051_P1 | chr15 | 28132134 | HERC2 | C | G | 0.37 | Missense |
| P051_P1 | chr15 | 28132164 | HERC2 | C | A | 0.55 | Missense |
| P051_P1 | chr15 | 28132249 | HERC2 | G | A | 0.55 | Nonsense |
| P051_P1 | chr15 | 28141473 | HERC2 | C | T | 0.19 | Missense |
| P051_P1 | chr15 | 28141475 | HERC2 | A | T | 0.44 | Missense |
| P051_P1 | chr15 | 28141574 | HERC2 | C | T | 0.3 | Missense |
| P051_P1 | chr15 | 28176726 | HERC2 | C | A | 0.34 | Missense |
| P051_P1 | chr15 | 28265861 | HERC2 | T | C | 0.31 | Missense |
| P051_P1 | chr15 | 34932328 | AQR | A | T | 0.5 | Missense |
| P051_P1 | chr15 | 40728799 | RAD51 | G | A | 0.47 | Missense |
| P051_P1 | chr15 | 43408972 | TP53BP1 | G | A | 0.38 | Missense |
| P051_P1 | chr15 | 43415789 | TP53BP1 | G | A | 1.17 | Missense |
| P051_P1 | chr15 | 43422015 | TP53BP1 | C | T | 0.43 | Missense |
| P051_P1 | chr15 | 43432204 | TP53BP1 | A | G | 0.35 | Missense |
| P051_P1 | chr15 | 43432592 | TP53BP1 | G | A | 0.42 | Nonsense |
| P051_P1 | chr15 | 43479975 | TP53BP1 | G | A | 0.32 | Missense |
| P051_P1 | chr16 | 23621465 | PALB2 | G | A | 0.51 | Nonsense |
| P051_P1 | chr16 | 23623027 | PALB2 | T | C | 0.62 | Missense |
| P051_P1 | chr16 | 56758588 | NUP93 | G | T | 0.15 | Missense |
| P051_P1 | chr16 | 56758653 | NUP93 | C | T | 0.27 | Nonsense |
| P051_P1 | chr16 | 89738704 | FANCA | A | G | 0.46 | Missense |
| P051_P1 | chr16 | 89742847 | FANCA | C | A | 0.21 | Nonsense |
| P051_P1 | chr17 | 7674944 | TP53 | C | A | 0.31 | Missense |
| P051_P1 | chr17 | 39471723 | CDK12 | C | T | 0.32 | Missense |
| P051_P1 | chr17 | 39501356 | CDK12 | G | T | 0.47 | Missense |
| P051_P1 | chr17 | 39526152 | CDK12 | C | A | 0.23 | Missense |
| P051_P1 | chr17 | 39530739 | CDK12 | C | T | 0.25 | Missense |
| P051_P1 | chr17 | 43091592 | BRCA1 | C | A | 0.51 | Missense |
| P051_P1 | chr19 | 45352798 | ERCC2 | G | A | 0.19 | Missense |
| P051_P1 | chr19 | 45355705 | ERCC2 | C | A | 0.18 | Missense |
| P051_P1 | chr19 | 45357512 | ERCC2 | C | A | 0.17 | Missense |
| P051_P1 | chr19 | 45357524 | ERCC2 | C | T | 0.18 | Missense |
| P051_P1 | chr19 | 45370225 | ERCC2 | C | T | 0.48 | Missense |
| P051_P1 | chr19 | 50402295 | POLD1 | T | C | 0.22 | Missense |
| P051_P1 | chr19 | 50403572 | POLD1 | T | C | 0.52 | Missense |
| P051_P1 | chr19 | 50408808 | POLD1 | C | A | 0.29 | Missense |
| P051_P1 | chr19 | 50408847 | POLD1 | C | T | 0.17 | Missense |
| P051_P1 | chr19 | 50409162 | POLD1 | G | T | 0.22 | Nonsense |
| P051_P1 | chr20 | 63667477 | RTEL1 | C | A | 0.25 | Missense |
| P051_P1 | chr22 | 41176353 | EP300 | C | T | 0.85 | Missense |
| P051_P1 | chr22 | 41177023 | EP300 | G | A | 0.54 | Missense |
| P051_P1 | chr22 | 41177890 | EP300 | C | A | 0.25 | Missense |
| P051_P1 | chr22 | 41177907 | EP300 | C | T | 0.18 | Nonsense |
| P051_P1 | chr22 | 41178120 | EP300 | C | T | 0.48 | Nonsense |
| P051_P1 | chr22 | 41178718 | EP300 | C | A | 0.14 | Missense |
| P051_P1 | chr22 | 41178745 | EP300 | G | T | 0.12 | Missense |
| P051_P1 | chr22 | 41178784 | EP300 | C | A | 0.21 | Missense |
| P051_P1 | chrX | 53382358 | SMC1A | T | C | 0.62 | Missense |
